# Cancer-associated fibroblasts facilitate premetastatic niche formation through lncRNA SNHG5-mediated angiogenesis and vascular permeability in breast cancer

**DOI:** 10.7150/thno.74753

**Published:** 2022-10-17

**Authors:** Huan Zeng, Yixuan Hou, Xinyue Zhou, Lei Lang, Haojun Luo, Yan Sun, Xueying Wan, Taixian Yuan, Rui Wang, Yongcan Liu, Rui Tang, Shaojie Cheng, Ming Xu, Manran Liu

**Affiliations:** 1 Key Laboratory of Laboratory Medical Diagnostics, Chinese Ministry of Education, Chongqing Medical University, Chongqing 400016, China.; 2 Experimental Teaching & Lab Management Center, Chongqing Medical University, Chongqing 400016, China.; 3 Department of Breast and Thyroid Surgery, the Second Affiliated Hospital of Chongqing Medical University, Chongqing 400010, China.; 4 Department of Cell Biology and Medical Genetics, Basic Medical School, Chongqing Medical University, #1 Yi-Xue-Yuan Rd., Yu-zhong District, Chongqing 400016, China.

**Keywords:** Cancer-associated fibroblasts, premetastatic niche, angiogenesis, vascular permeability, lncSNHG5

## Abstract

**Background:** Metastasis is the leading cause of death in patients with breast cancer (BC). Primary tumors create a premetastatic niche (PMN) in secondary organs for subsequent metastases. Cancer-associated fibroblasts (CAFs) are a predominant stromal component in the tumor microenvironment and serve as a major contributor to tumor metastasis. However, the function and mechanism of primary CAFs in the premetastatic niche of secondary organs remain unclear in BC.

**Methods:** We investigated the expression profiles of lncRNAs in pairs of CAFs and NFs derived from breast tumor tissues using lncRNA microarray. The expression levels of lncSNHG5, ZNF281, IGF2BP2, CCL2 and CCL5 were assessed by qRT-PCR; the protein levels of related genes (e.g., ZNF281, IGF2BP2, CCL2, and CCL5) were analyzed using western blotting and/or ELISA in primary and immortalized CAFs and clinical samples. Tubule formation and three-dimensional sprouting assays and tissue fluorescence staining were conducted to investigate angiogenesis. *In vitro* permeability assays, trans-endothelial invasion assays, *in vivo* permeability assays and tissue fluorescence staining were conducted to examine vascular permeability. The regulatory mechanism of lncSNHG5 was investigated by RNA sequencing, fluorescent *in situ* hybridization, cellular fractionation assay, mass spectrometry, RNA pull-down, RNA immunoprecipitation, gene-specific m6A assay, chromatin immunoprecipitation, dual luciferase reporter assay and actinomycin D treatment in CAFs and NFs.

**Results:** LncSNHG5 was highly expressed in breast CAFs and played an essential role in premetastatic niche formation by promoting angiogenesis and vascular leakiness through regulation of ZNF281 in CAFs. lncSNHG5 enhanced ZNF281 mRNA stability by binding with the m6A reader IGF2BP2. Enhanced ZNF281 transcriptionally regulated CCL2 and CCL5 expression to activate P38 MAPK signaling in endothelial cells. High CCL2 and CCL5 expression was associated with tumor metastasis and poor prognosis in BC patients. The inhibitors RS102895, marasviroc and cenicriviroc inhibited angiogenesis and vascular permeability in the PMN by blocking the binding of CCL2/CCR2 and CCL5/CCR5. The lncSNHG5-ZNF281-CCL2/CCL5 signaling axis plays an essential role in inducing premetastatic niche formation to promote BC metastasis.

**Conclusions:** Our work demonstrates that lncSNHG5 and its downstream signaling ZNF281-CCL2/CCL5 in CAFs play a crucial role in premetastatic niche formation in breast cancer and may serve as potential targets for the diagnosis and treatment of BC metastasis.

## Introduction

Breast cancer (BC) is one of the most common malignancies and the second leading cause of cancer mortality among women worldwide 1. With rapid advances in BC treatment 2, better clinical outcomes have been achieved. Nevertheless, metastasis is still the main cause of death for BC patients, with a median overall survival of only 2-3 years 3. The premetastatic niche (PMN) created by primary tumors in a specific secondary organ is crucial to the subsequent colonization and metastasis of tumor cells, including vascular permeability and angiogenesis, immunosuppression, inflammation, reprogramming and organotropism 4. Moreover, the initial stages of PMN formation are characterized by angiogenesis and vascular permeability 5. The function and mechanism of blood vessels in primary tumor sites have been well studied, but little is known about the changes in blood vessels in secondary metastasis sites. Several PMN biomarkers related to angiogenesis and vascular permeability have been reported to aid in the diagnosis and treatment of metastatic cancer 6-9. However, the mechanisms regulating PMN formation are complex and have their own characteristics in different tumors. Therefore, exploring the mechanism of PMN formation in a tumor may provide useful insights and valuable strategies for the treatment of metastatic cancer.

Cancer-associated fibroblasts (CAFs), the major stromal component of the tumor microenvironment, are critical to tumor development 10. Our and other previous studies have shown that CAFs can promote cancer growth and metastasis by secreting cytokines and exosomes, synthetizing and remodeling the extracellular matrix, and metabolic remodeling in the primary tumor microenvironment 11-15. CAFs are also major players in angiogenesis by secreting cytokines, including VEGFA, PDGFC, SDF1, WNT2, and WNT5a 16-19. However, little is known about whether primary CAFs can affect metastasis in distant organs. Recently, CAFs have been reported to increase the permeability of lymphatic and brain endothelial cells to facilitate cholangiocarcinoma cells and BC cells crossing the vascular endothelial layer, leading to colonization in the brain 20, 21. Another interesting study showed that primary CAFs can deliver integrin β1-positive extracellular vesicles (EVs) to lung fibroblasts to cause lung metastasis 22. These studies indicate that CAFs in the primary tumor can affect metastatic formation, but the details remain unknown.

Long non-coding RNAs (lncRNAs) refer to RNAs that are longer than 200 nucleotides and exhibit non-encoding functions 23. Accumulating evidence over the past decade has indicated that lncRNAs are implicated in multiple pathophysiological processes, including proliferation, metastasis, drug resistance, stemness, metabolism, immune escape and angiogenesis 24-26. LncRNAs specifically interact with DNA, RNA, and proteins depending on their localization to modulate chromatin function, regulate cytoplasmic mRNA stability and translation, or interfere with signaling pathways 27. However, the underlying functions and mechanisms of stromal lncRNAs in CAFs remain poorly understood.

In this study, we identified a significantly upregulated lncSNHG5 in primary breast CAFs, which plays an essential role in PMN formation via vascular leakage and angiogenesis. lncSNHG5 can interact with IGF2BP2 to augment the stability of ZNF281 mRNA in an m6A-dependent manner. The enhanced ZNF281 transcriptionally regulates CCL2 and CCL5 expression in CAFs, which then activates P38 MAPK signaling in endothelial cells to induce PMN formation in the metastatic environment.

## Material and Methods

### Clinical samples

The human paired breast cancer tissue and paracancerous tissue used in this study were collected from breast cancer patients, all of whom had not received chemotherapy or radiotherapy at the First Affiliated Hospital and the Second Affiliated Hospital of Chongqing Medical University. The investigation was approved by the Ethics Committee of Chongqing Medical University.

### Isolation, immortalization and cell culture of stromal fibroblasts

Cancer-associated fibroblasts (CAFs) and their corresponding normal fibroblasts (NFs) were isolated from BC tissues and immortalized, as described in our previous study 28. CAFs and NFs were cultured in DMEM (Gibco, Australia) containing 10% fetal bovine serum (FBS; Gibco, Australia) and placed in incubators containing 5% CO_2_ at 37 °C. Human umbilical vein endothelial cells (HUVECs), human normal mammary epithelial cells (MCF-10A) and breast cancer cells (SK-BR-3, MCF-7, Hs578T, BT549 and MDA-MB-231) were purchased from the American Type Culture Collection (ATCC). The above cells were all cultured in RPMI 1640 medium (Gibco, Australia) with 10% FBS in a 5% CO_2_ standard humidified incubator at 37 °C.

### LncRNA microarray and RNA sequencing

LncRNAs in CAFs and NFs were analyzed using the Agilent SBC human lncRNA Microarray V5 (4*180K), which was performed by Shanghai Biotechnology Corporation. The microarray data are presented in the Supplementary File.

Total RNA was extracted from NFs/Ctrl and NFs/lncSNHG5. Then, RNA was purified through rRNA depletion and subsequently used for cDNA synthesis and RNA amplification. Next, random hexamer primer cDNA libraries were sequenced on the DNBSEQ-T7 sequencing platform according to the manufacturer's instructions for paired-end 150 bp reads (Lifegenes, Shanghai, China). HTSeq v0.13.5 was used to count the read numbers mapped to each gene. Differential expression analysis of two samples was performed using the DESeq2 R package (1.26.0). The raw RNA-seq data were submitted to the Sequence Read Archive database (SRA; Identifiers: SRP392164).

### Bioinformatics analyses

For Gene Ontology (GO) enrichment analysis, differentially expressed genes were analyzed using the clusterProfiler R package (v3.12.0). For Kyoto Encyclopedia of Genes and Genomes (KEGG) enrichment analysis, KOBAS v3.0 software was implemented to test the statistical enrichment of differentially expressed genes in KEGG pathways. GO or KEGG terms with P values less than 0.05 were considered significantly enriched by differentially expressed genes. GSEA was performed using GSEA 4.0.3 software and the functional gene sets defined by GSEA websites (http://www.gsea-msigdb.org/gsea/downloads.jsp) to analyze the TCGA-BRCA dataset according to the levels of gene expression.

### Reagents, RNA interference and plasmids

The following reagents were used in our work: rCCL2 and rCCL5 (R&D Systems, USA), axitinib (Selleck, USA), RS102895 (Selleck, USA), maraviroc (Selleck, USA), cenicriviroc (Selleck, USA), and SB203580 (Selleck, USA).

To construct the engineered cells with stable knockdown or overexpression of target genes, the lentivirus carrying shRNA against lncSNHG5, ZNF281, IGF2BP2, CCL2 and CCL5 and lentiviral lncSNHG5-, ZNF281-, IGF2BP2-, CCL2- and CCL5-overexpressing constructs and their controls were infected into CAFs or NFs according to the manufacturer's instructions. The shRNA sequences are provided in Table S1.

Full-length and deletion mutants of IGF2BP2 (FL, T1, T2 and T3) were amplified by PCR and inserted into the pcDNA3.1-Flag-tagged plasmid. The 5′-UTR, CDS, and 3′-UTR of ZNF281 were cloned into the pcDNA3.1 vector with biotin RNA labeling. The primer sequences used for plasmid construction are listed in Table S2.

### RNA preparation and qRT-PCR

Total RNA was extracted with TRIzol and then reverse transcribed into cDNA using a PrimeScript RT Reagent Kit (TaKaRa, China). A SYBR Premix Ex Taq™ II Kit (TaKaRa, China) was used to perform quantitative real-time PCR for the amplification of cDNA. The relative gene expression level was measured by the 2-ΔΔCT method and normalized to β-actin expression. The primer sequences used for qRT-PCR are listed in Table S3.

### Western blotting and ELISAs

Total protein was extracted by lysing whole cells or tissues in RIPA lysis buffer (Boster, China), and the protease inhibitor PMSF (Boster, China) was added to prevent protein degradation. A total of 50-80 μg protein was separated using 8-12% SDS-PAGE gel and electrically transferred to 0.22 μM PVDF membrane (Bio-Rad, USA), which was blocked with 5% nonfat milk at room temperature for 3 h and then incubated at 4 °C overnight with the special primary antibodies: Occludin (Abcam, ab216327, 1:1000), ZO-1 (Cell Signaling, #8193, 1:1000), ZNF281 (SAB, #43566, 1:1000), IGF2BP2 (Proteintech, 11601-1-AP, 1:2000), VEGFA (Abcam, ab1316, 1:1000), CCL2 (SAB, #45065, 1:500), CCL5 (SAB, #32935, 1:500), p-AKT (CST, #12694, 1:1000), AKT (CST, #9272, 1:1000), p-STAT3 (CST, #9131, 1:500), STAT3 (CST, #4904, 1:500), p-P38 (CST, #4631, 1:500), P38 (CST, #9212, 1:1000), p-VEGFR2 (Sangon, D155165, 1:800), VEGFR2 (Sangon, D151118, 1:800), and β-Actin (Biosharp, 1:1000). The PVDF membranes were incubated with HRP-labeled secondary antibody for 1.5 h at room temperature, followed by visualization of the protein bands using an enhanced chemiluminescence system (Amersham Pharmacia Biotech) with ECL detection reagents (Bio-Rad, USA). Protein concentrations of CCL2, CCL5 and VEGFA were measured in CM derived from CAFs or NFs or serum from healthy donors and BC patients using ELISA kits (R&D, USA) following the manufacturer's instructions.

### Fluorescence *in situ* hybridization (FISH)

The cellular localization of lncSNHG5 in CAFs was detected using a fluorescence *in situ* hybridization kit and FISH probe (GenePharma, China) following the manufacturer's protocol. Next, CAFs were incubated with Cy3-labeled lncSNHG5 probes at 4 °C overnight, after which DAPI was utilized to stain the nuclei. Images were taken using a confocal microscope.

### Nuclear and cytoplasmic fractionation

Cytoplasmic and nuclear RNA from CAFs was extracted using the PARIS^TM^ Kit (Invitrogen, USA). GAPDH mRNA and U6 RNA were used as internal controls for cytoplasmic and nuclear RNA, respectively. Cytoplasmic and nuclear RNA were further quantified by qRT‐PCR.

### Immunohistochemistry (IHC)

The paraffin-embedded tumor tissues obtained from nude mice and breast cancer patients were sectioned at a thickness of 4 µm. After being deparaffinized with xylene, dehydrated, and incubated with 10% goat serum for antigen retrieval, the tissue sections were subsequently incubated with CD31 (Abcam, ab9498, 1:100) and ZNF281 (SAB, #43566, 1:200) primary antibodies for 1 h and then with HRP secondary antibody for 20 min at room temperature. Diaminobenzidine (DAB) was used to stain the tissue sections. The IHC images were visualized and captured under an Eclipse 80i microscope (Nikon, Japan).

### Preparation of conditioned medium (CM)

CAFs or NFs (1×10^6^) were plated in a 6-well plate. The growth medium with serum was replaced by 1 mL of serum-free medium, and the cells were further cultured for nearly 30 h. Following centrifugation at 500 rpm for 5 min, the collected supernatant was used as CM for subsequent experiments.

### Three-dimensional sprouting and tube formation assays

For tube formation assays, HUVECs were plated in a 96-well plate precoated with growth factor-deprived Matrigel and cultured in 150 µL CM for 6 h. Images were captured using a microscope and analyzed using ImageJ. For three-dimensional sprouting assays, 1×10^3^ HUVECs were cultured in 0.24% carboxymethylcellulose medium in round-bottom 96-well plates for 24 h. Spheroids of suspended endothelial cells were collected by centrifugation and resuspended with collagen type I (Corning, USA) at a concentration of 2 mg/mL. Then, the three-dimensional collagen gels were incubated with the indicated CM for more than 24 h. The sprouting capacity of HUVECs was assessed by counting the number of branches under a microscope with at least 10 spheroids per group.

### *In vitro* permeability assays and trans-endothelial invasion assays

For *in vitro* permeability assays, HUVECs were treated with supernatant from CAFs or NFs for 48 h to form an endothelial monolayer, and then rhodamine B-dextran was added to the upper layer of the transwell filters (0.4 μm, Corning). After 30 min, the medium in the lower layer of the chamber was collected to measure the fluorescence intensity. The fluorescence was detected at 544 nm excitation and 590 nm emission. For trans-endothelial invasion assays, 2×10^4^ GFP-labeled Hs578T and MDA-MB-231 cells were seeded into transwell filters (8 μm, BD Biosciences) with endothelial cell monolayers pretreated with CM. After 14 h, the Hs578T or MDA-MB-231 cells in the upper layer of the chamber were removed, while those labeled with GFP in the lower layer were observed and counted under a fluorescence microscope.

### RNA stability assays

CAFs or NFs were treated with actinomycin D (10 µg/mL) to block mRNA transcription. Total RNA was extracted with TRIzol at the designated time. The mRNA levels of ZNF281 were evaluated using qRT-PCR, and β-actin served as the internal reference.

### RNA pulldown and RNA immunoprecipitation (RIP) assays

Full-length antisense or sense lncSNHG5 was transcribed using a T7 *in vitro* Transcription Kit (Roche, USA) following the manufacturer's procedures. Biotinylated lncSNHG5 or ZNF281, which was bound to streptavidin-labeled agarose beads (Invitrogen), was incubated with whole-cell lysates of CAFs. Then, the bead-bound complexes of RNA and protein were carefully eluted with elution buffer. The eluted proteins were used for silver staining and mass spectrometry or WB.

For antisense DNA probe pull-down assays, antisense or sense biotin-labeled DNA probes specifically against lncSNHG5 were incubated with CAF lysates for 2 h. Streptavidin-coupled agarose beads were added to separate the RNA-protein or RNA-RNA compound, and western blotting or qRT-PCR was then performed. The primer sequences used in the pull-down assay can be found in Table S4.

RIP assays were conducted using an RNA-binding protein immunoprecipitation kit (Millipore, USA) based on the manufacturer's protocols. Briefly, 3×10^7^ CAFs were lysed in lysis buffer at 4 °C, and the magnetic beads were incubated with 5 µg of IGF2BP2 antibody for 1 h at room temperature. Then, the cell lysate was incubated with the antibody-bead complex in immunoprecipitation buffer overnight at 4 °C. The IGF2BP2-RNA immunoprecipitates binding with magnetic beads were eluted using elution buffer for 0.5 h at 55 °C. RNA was purified and then used for qRT-PCR.

### Luciferase reporter and chromatin immunoprecipitation (ChIP) assays

For the luciferase reporter assay, the ZNF281 promoters, CCL2 and CCL5 promoters with ZNF281 binding motifs (WT) or their mutant promoters were inserted into the pGL3 vector (Promega). The pGL3-ZNF281-promoter-WT, pGL3-CCL2-promoter (WT or Mut) and pGL3-CCL5-promoter (WT or Mut) were transfected into CAFs with the pCMV-Renilla control. The luciferase activity was measured approximately 36 h after transfection.

ChIP assays were performed using the Chromatin IP Kit (CST, #9005, USA). Briefly, CAFs were fixed with 1% formaldehyde and glycine to cross-link histones and nonhistone proteins to chromatin. The anti-ZNF281 antibody or corresponding IgG was used to pull down the chromatin associated with ZNF281. Subsequently, the specific DNA fragments were quantified by qRT-PCR. The primer sequences used for the ChIP assay are listed in Table S5.

### Gene-specific m6A qPCR assays

Gene-specific m6A qPCR assays were performed using the Magna MeRIP m6A Kit (Millipore, Billerica). In short, 100 µg of total RNA was sheared by metal ion-induced fragmentation to approximately 200 nt in length, and the sheared RNAs were purified and incubated with the beads combined with m6A or IgG antibody in IP buffer (500 µL, 1×) overnight at 4 °C. Then, the methylated ZNF281 mRNA was eluted from the bead-bound immunoprecipitation complex. The MeRIP RNA was further quantified by qRT-PCR, and one-tenth of fragmented RNA was kept as an input control. The relative levels of m6A in each group were normalized to the tenfold input.

### Tissue fluorescence staining

Tissue sections were first blocked with a 3% hydrogen peroxide solution to block endogenous peroxidase, then blocked with BSA, incubated overnight with primary antibody CD31, incubated with HRP secondary antibody at room temperature for 50 min, and reacted with TYR-520 fluorescent dye in the dark for 10-15 min. These sections were reincubated with the primary antibodies ZO-1 and Occludin, and the above steps were repeated, switching to TYR-570 and TYR-690 fluorescent dyes. Fluorescence scanning was performed after DAPI staining.

### Xenograft Mouse Model

Orthotopic tumor xenografts were established as described previously 13. MDA-MB-231 cells (1×10^6^ cells/mouse) were mixed with an equivalent number of NFs/Ctrl, CAFs/shNC, CAFs/sh lncSNHG5, CAFs/shZNF281, CAFs/sh lncSNHG5/ZNF281, CAFs/RS102895, CAFs/Maraviroc or CAFs/Cenicriviroc in 200 µl PBS:Matrigel at a ratio of 1:1. The cell mixture was subcutaneously injected into 4-week-old female nude mice (n=10 for each group). Mice were fed RS102895, maraviroc or cenicriviroc every 3 days by gavage. For the *in vivo* permeability assay, 100 μg/g rhodamine B isothiocyanate-dextran was intravenously administered to nude mice for 3 h, followed by cardiac perfusion to remove excess dye. After 2 weeks, the mice (6 weeks old) were sacrificed for tissue collection and assessment. The lungs of the mice were used for frozen sections and fluorescent staining with CD31, ZO-1 and Occludin antibodies, and the blood of the mice was used to measure the serum levels of CCL2 and CCL5. Six weeks later, the nude mice (10 weeks old) were sacrificed. The mouse lungs were stained with hematoxylin and eosin to detect lung metastases. All animal experiments were approved by the animal care ethics committees of Chongqing Medical University.

### Statistical Analysis

Statistical analysis was performed using GraphPad Prism 8.0 software. Differences between groups were compared using paired/unpaired t test, a chi-square test, or one-way ANOVA. Kaplan‒Meier analysis was conducted to assess the rate of overall survival and relapse-free survival. The data are presented as the means ± SDs from the data of experiments that were independently repeated at least three times. A value of P<0.05 was considered statistically significant.

## Results

### LncSNHG5 is highly expressed in breast CAFs and closely related to tumor metastasis

**Figure 1:**
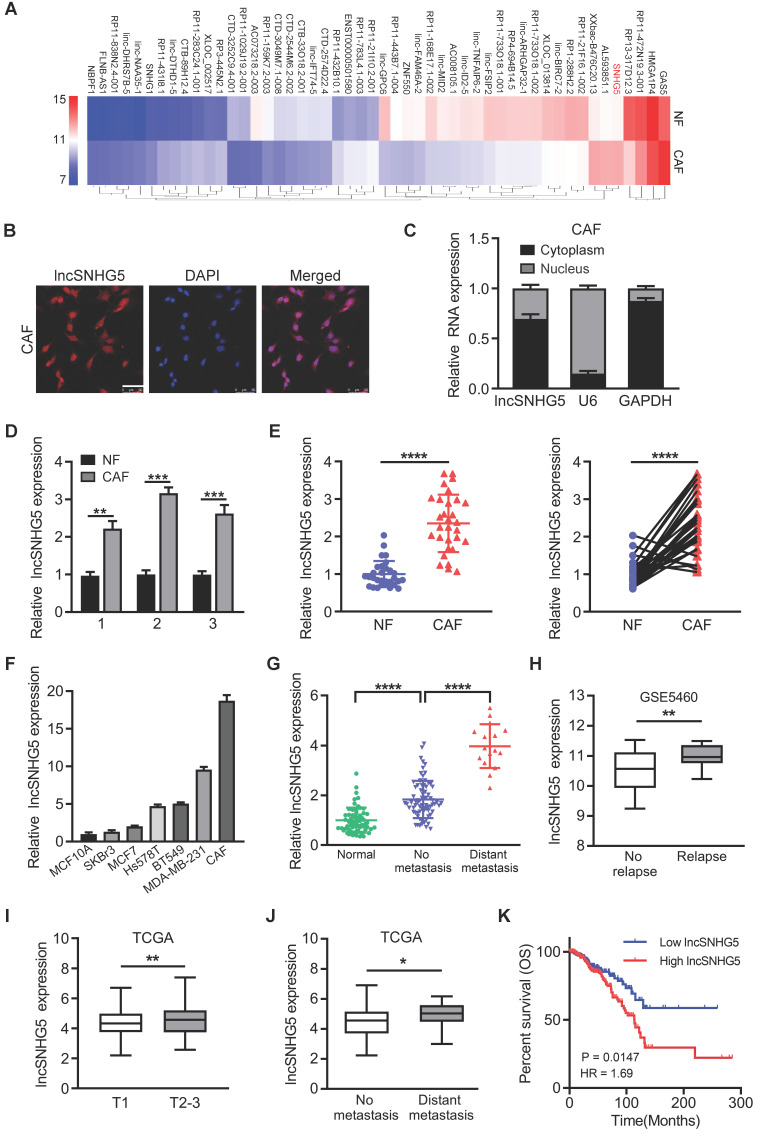
** High expression of LncSNHG5 in breast CAFs is closely related to breast cancer malignancy. (A)** LncRNA expression profiles in paired NFs and CAFs are shown in a heatmap. **(B)** Representative images of lncSNHG5 localization in CAFs were visualized using an RNA-FISH assay (scale bar, 50 µm). **(C)** lncSNHG5 fractionation in CAFs was assessed using qRT-PCR. GAPDH or U6 served as a positive control for cytoplasmic gene or nuclear gene expression, respectively. **(D-E)** The relative lncSNHG5 expression was tested in 3 paired immortalized CAFs and NFs (D) and 30 paired primary CAFs and NFs isolated from BC tissues (E) using qRT-PCR. **(F)** The relative expression of lncSNHG5 was measured using qRT-PCR in CAFs and breast cancer cells. **(G)** qRT-PCR to detect lncSNHG5 expression in human breast tumors with (16 cases) or without (76 cases) metastasis and adjacent normal tissues (60 cases). **(H)** The expression of lncSNHG5 was analyzed in recurrent and nonrecurrent breast tumors using GSE5460 data. **(I-J)** The correlation between the expression level of lncSNHG5 and advanced tumor stage (T stage) and distant metastasis was analyzed based on TCGA BRCA dataset. **(K)** Kaplan-Meier survival analysis was used to evaluate the overall survival of BC patients according to lncSNHG5 expression in TCGA data. The data represent the mean±SD (*P < 0.05, **P < 0.01, ***P < 0.001).

To identify the differentially expressed lncRNAs in CAFs, we performed a lncRNA microarray using primary NFs and CAFs isolated from BC tissues. The top 50 dysregulated lncRNAs are shown in a heatmap (Figure 1A). To confirm the aberrantly enhanced lncRNAs identified by microarray, ten of the upregulated lncRNAs were randomly selected and verified using qRT-PCR in paired CAFs and NFs (Figure S1A), among which lncSNHG5 was the most significantly upregulated. We confirmed that lncSNHG5 was a cytoplasmic RNA in breast CAFs using RNA-FISH (Figure 1B) and nucleocytoplasmic fractionation assays (Figure 1C). To understand the potential relationship between the expression levels of lncSNHG5 and the clinical characteristics of breast tumors, we performed qRT-PCR in 3 pairs of immortalized and 30 pairs of primary NFs and CAFs isolated from breast tumors, the results of which showed that the expression of lncSNHG5 was stably increased in CAFs (Figure 1D-E). Moreover, the lncSNHG5 expression levels of CAFs were significantly higher than those of breast cancer cells (SK-BR-3, MCF-7, Hs578T, BT549 and MDA-MB-231) and normal breast epithelial cells (MCF-10A; Figure 1F). Next, we evaluated the levels of lncSNHG5 in human breast tumor tissues with or without metastasis and their adjacent normal tissues and found that lncSNHG5 expression was higher in breast tumor tissues than in normal tissues, and that it was highly expressed in breast tumors with metastasis compared to those without metastasis (Figure 1G). After analysis of the GEO dataset GSE5460 (n=64), enhanced lncSNHG5 was positively correlated with tumor recurrence (Figure 1H). In addition, data analysis from The Cancer Genome Atlas (TCGA) further confirmed that the increased lncSNHG5 was positively related to T stage, distant metastasis and a poor prognosis (Figure 1I-K). Finally, we assessed the relationship between lncSNHG5 levels and clinical pathological characteristics in 92 BC patients. Increased lncSNHG5 was closely related to lymph node metastasis, distant metastasis and TNM stage (Table S6). In summary, lncSNHG5 is highly expressed in CAFs and closely related to BC malignancy.

To investigate the biological function of lncSNHG5 in CAFs, we overexpressed ectopic lncSNHG5 in NFs (Figure S1B) and compared the differentially expressed genes between NFs-NC and NFs-lncSNHG5 using RNA-seq. We identified more than one thousand dysregulated genes by setting a threshold value of “P < 0.05 and fold change ≥1.5”. As visualized by the volcano plot, 621 upregulated and 528 downregulated genes were found to be differentially expressed between NFs/NC and NFs/lncSNHG5 (Figure S1C). The top 50 dysregulated genes are shown in the heatmap (Figure S1D). Interestingly, after analysis by bioinformatics, lncSNHG5 was found to be potentially involved in the regulation of angiogenesis (Figure S1E) and cell migration (Figure S1F), and KEGG enrichment results also indicated that lncSNHG5 was mainly related to cytokine-related biological processes (Figure S1G), suggesting that lncSNHG5 in stromal fibroblasts potentially affects tumor metastasis by regulating angiogenesis and cytokine-related biological processes in breast tumors.

### LncSNHG5 in breast CAFs primes a premetastatic niche by inducing angiogenesis and vascular leakiness

**Figure 2:**
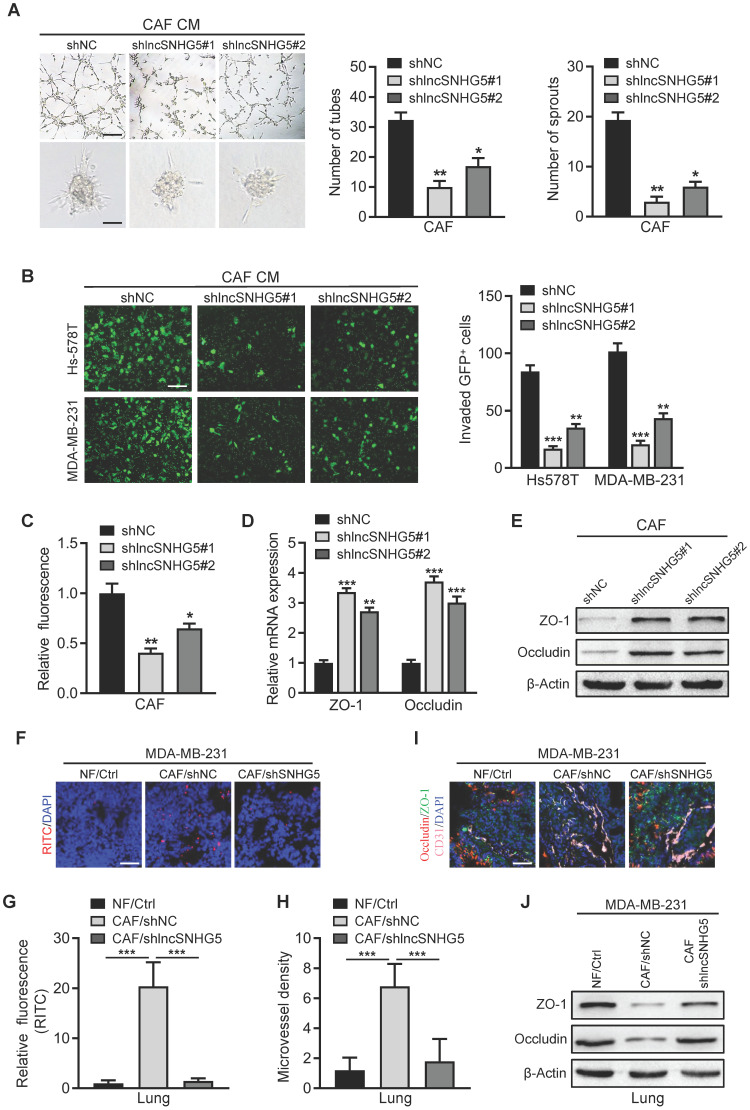
**LncSNHG5 in breast CAFs primes the premetastatic niche by inducing angiogenesis and vascular leakiness. (A)** Tube formation (upper panel) and spheroid sprouting (bottom panel) of HUVECs were measured after treatment with CM from lncSNHG5-knockdown CAFs and control CAFs (scale bar, 100 µm). **(B-C)** CM from lncSNHG5-knockdown CAFs or control CAFs was applied to construct HUVEC monolayers, and the invasive GFP^+^ tumor cells across the HUVEC monolayers were assessed by transwell assay (B). The permeability of HUVEC monolayers was tested by rhodamine-dextran (70 kDa) staining (C). **(D-E)** HUVECs were cultured in CM derived from lncSNHG5-knockdown and control CAFs for 30 h, and the mRNA and protein expression levels of ZO-1 and Occludin in HUVECs were examined using qRT-PCR and WB. **(F)** MDA-MB-231 cells mixed with NFs/Ctrl, CAFs/shNC or CAFs/shlncSNHG5 were orthotopically injected into nude mice for 2 weeks. The mice were injected with rhodamine-dextran before lung metastasis, and the vascular permeability of mouse lungs is shown (scale bar, 100 µm). **(G)** The fluorescence levels of rhodamine-dextran in mouse lungs were calculated using ImageJ software and normalized to DAPI levels. **(H)** The microvessel density in mouse lungs was quantified by counting the microvessel numbers under a fluorescence microscope. **(I)** Representative images of fluorescent staining of ZO-1 (green), Occludin (red) and CD31 (pink) in lung tissue are shown (scale bar, 100 µm), and the vascular structures were labeled using CD31 (pink). **(J)** The protein levels of ZO-1 and Occludin in mouse lung tissues before metastasis were assessed by western blotting. The data represent the mean ± SD (*P < 0.05, **P < 0.01, ***P < 0.001).

Our and other previous studies have shown that CAFs in primary tumors play a crucial role in lung metastasis of breast cancer 11, 29, and premetastatic niche (PMN) formation is considered to be an important step for tumor metastasis in distant organs 30. We next asked whether CAFs in primary tumors contribute to PMN formation. Active angiogenesis and increased vascular permeability are initial steps and key features of premetastatic niche formation 31; therefore, we explored whether lncSNHG5 regulates angiogenesis and vascular permeability to prime the premetastatic niche. When lncSNHG5 was knocked down in CAFs using two independent lentiviruses (Figure S2A), the conditioned medium (CM) derived from lncSNHG5-knockdown CAFs dramatically decreased the tube formation and sprouting of HUVECs (Figure 2A). In contrast, CM from lncSNHG5-overexpressing NFs clearly increased the tube formation and sprouting of HUVECs (Figure S2B-C), supporting the idea that lncSNHG5 potentially regulates angiogenesis. To understand whether lncSNHG5 can regulate endothelial permeability, trans-endothelial invasion assays and *in vitro* permeability assays were then carried out. Compared with the control, CM from lncSNHG5-knockdown CAFs markedly dampened endothelial permeability (Figure 2B-C), while CM from lncSNHG5-overexpressing NFs clearly promoted endothelial permeability (Figure S2D-E). Coculture of HUVECs with the supernatant from lncSNHG5-knockdown CAFs or lncSNHG5-overexpressing NFs showed that lncSNHG5 knockdown in CAFs markedly increased expression of tight junction biomarkers ZO-1 and Occludin in HUVECs (Figure 2D-E), whereas overexpression of lncSNHG5 in NFs clearly reduced ZO-1 and Occludin levels in HUVECs (Figure S2F-G).

To evaluate whether lncSNHG5 in CAFs plays a pivotal role in angiogenesis and vascular permeability in the premetastatic niche, a mixture of MDA-MB-231 and NFs or CAF/shNC or CAF/shSNHG5 was orthotopically injected into the mammary fat pad of nude mice to establish a premetastatic mouse model for 2 weeks. Rhodamine-dextran was intravenously injected into tumor-bearing mice to assess lncSNHG5-mediated vascular permeability *in vivo*, and fluorescent staining was performed to determine the levels of vascular markers CD31 and tight junction proteins ZO-1 and Occludin in lung tissue to reflect the angiogenesis and permeability changes of lung blood vessels. We observed that the permeability (Figure 2F-G) and microvessel density (MVD; Figure 2H) of pulmonary capillaries were significantly increased, and the ZO-1 and Occludin levels (Figure 2I-J) were clearly decreased in the tumor-bearing mice injected with MDA-MB-231 cells and CAFs (MDA-MB-231/CAFs shNC) before lung metastasis, while the lung capillary permeability and MVD were markedly decreased, and the expression levels of ZO-1 and Occludin were increased in the tumor-bearing mice injected with the mixture of MDA-MB-231 cells and NFs (MDA-MB-231/NFs Ctrl) or lncSNHG5-knockdown CAFs (MDA-MB-231/CAFs shlncSNHG5; Figure 2F-J). These data demonstrate that lncSNHG5 in breast CAFs can induce angiogenesis and vascular leakiness to promote premetastatic niche formation.

### LncSNHG5 promotes angiogenesis and endothelial permeability by regulating ZNF281 in CAFs

**Figure 3:**
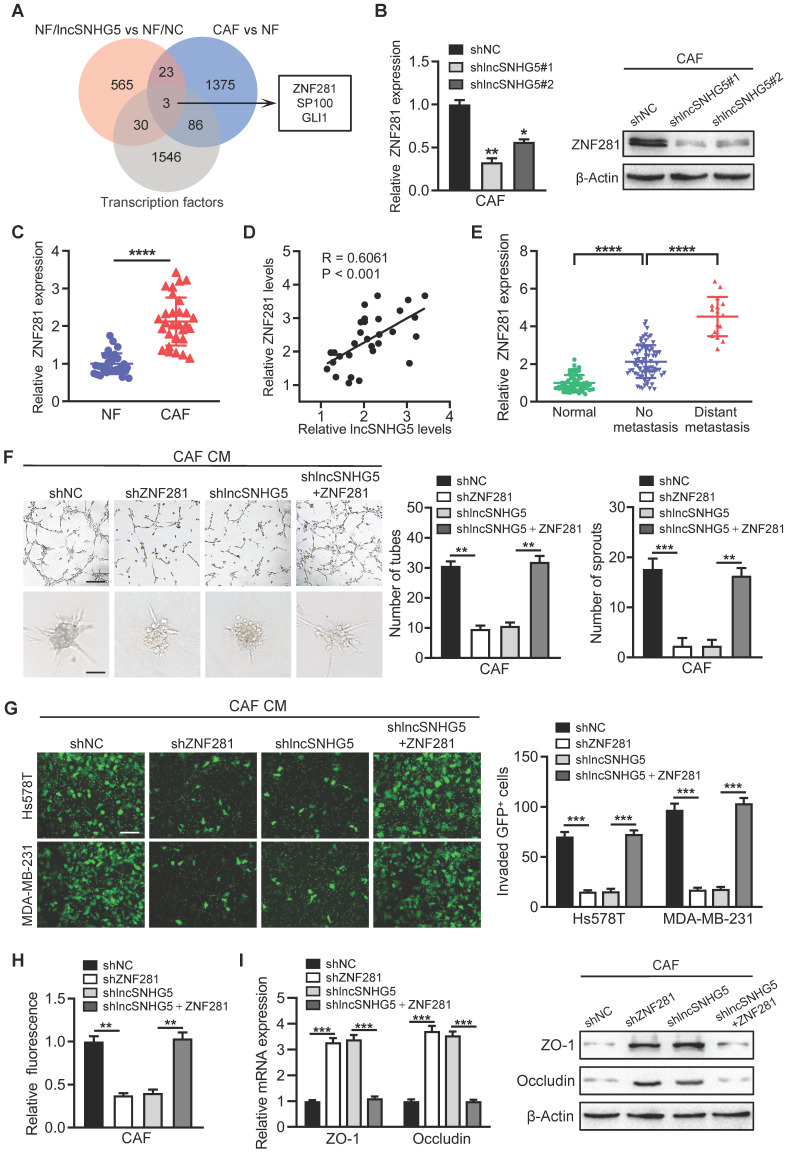
** LncSNHG5 promotes angiogenesis and endothelial permeability by regulating ZNF281 in CAFs. (A)** Venn diagrams were used to demonstrate the putative targets of lncSNHG5 in CAFs. **(B)** The expression of ZNF281 in control CAFs and lncSNHG5-knockdown CAFs was evaluated using qRT-PCR and WB. **(C)** qRT-PCR was used to evaluate the relative lncSNHG5 levels in 30 paired primary CAFs and NFs separated from BC tissues. **(D)** Pearson correlation of ZNF281 levels and lncSNHG5 levels in CAFs. **(E)** qRT-PCR was used to detect ZNF281 levels in human breast tumors with (16 cases) or without (76 cases) metastasis and adjacent normal tissues (60 cases). (F) Effect of CM derived from CAF/shNC, shZNF281, sh lncSNHG5 and sh lncSNHG5/ZNF281 on tube formation (upper panel) and sprouting spheroids (lower panel) of HUVECs (scale bar, 100 µm). **(G-H)** HUVECs were treated with CM from the above groups to form monolayers, and then the permeability of HUVECs was assessed by transwell assay with GFP-labeled MDA-MB-231 and Hs578T cells seeded in the upper chamber of the insert (G) or rhodamine-dextran fluorescence staining (H). **(I)** qRT-PCR and WB were used to check the expression of ZO-1 and Occludin in HUVECs cultured with CM from the indicated cells for 30 h. Data are presented as the mean ± SD (*P < 0.05, **P < 0.01, ***P < 0.001).

Transcription factors play a pivotal role in angiogenesis and vascular permeability 32, 33. To search for the potential target genes of lncSNHG5, we analyzed the upregulated genes with a cutoff value of “P < 0.05 and fold change ≥1.5” in the gene expression profile (NFs/lncSNHG5 vs. NFs/NC, and CAFs vs. NFs) and found that the transcription factors ZNF281, SP100 and GLI1 were potentially regulated by lncSNHG5 (Figure 3A). The mRNA and protein levels of ZNF281 were significantly elevated in lncSNHG5-overexpressing NFs (Figure S3A) but remarkably reduced in lncSNHG5-knockdown CAFs (Figure 3B). The increased ZNF281 in CAFs was verified by qRT-PCR in 30 pairs of primary breast NFs and CAFs (Figure 3C) and 3 pairs of immortalized NFs and CAFs (Figure S3B). Using Pearson correlation analysis, we further validated that ZNF281 levels were positively correlated with lncSNHG5 expression (Figure 3D), suggesting that ZNF281 is the target of lncSNHG5. Furthermore, ZNF281 was highly expressed in BC and was more highly expressed in metastatic breast tumors than in those without metastasis (Figure 3E). To understand whether ZNF281 is related to angiogenesis, we performed GSEA. The results showed that ZNF281 is positively related to endothelial cell migration and vasculature development (Figure S3C).

To test the role of ZNF281 in endothelial cells, we successfully established lentivirus-mediated ZNF281 knockdown CAFs and ectopic ZNF281-overexpressing NFs (Figure S3D-E). Compared with the control, CM from ZNF281-overexpressing NFs clearly facilitated tube formation and sprouting of HUVECs (Figure S3F-G) and endothelial permeability (Figure S3H-I) and suppressed the expression of ZO-1 and Occludin (Figure S3J). Conversely, knockdown of ZNF281 in CAFs dramatically dampened tube formation and sprouting of HUVECs (Figure 3F) and endothelial permeability (Figure 3G-H) and increased the expression of ZO-1 and Occludin in HUVECs (Figure 3I). Moreover, restoration of ZNF281 expression in lncSNHG5 knockdown CAFs partially rescued these aforementioned phenotypes and the levels of ZO-1 and Occludin in HUVECs (Figure 3F-I). Thus, these data demonstrate that lncSNHG5 can promote angiogenesis and endothelial permeability by regulating ZNF281 in CAFs.

### lncSNHG5 enhances ZNF281 mRNA stability by binding with the m6A reader IGF2BP2

**Figure 4:**
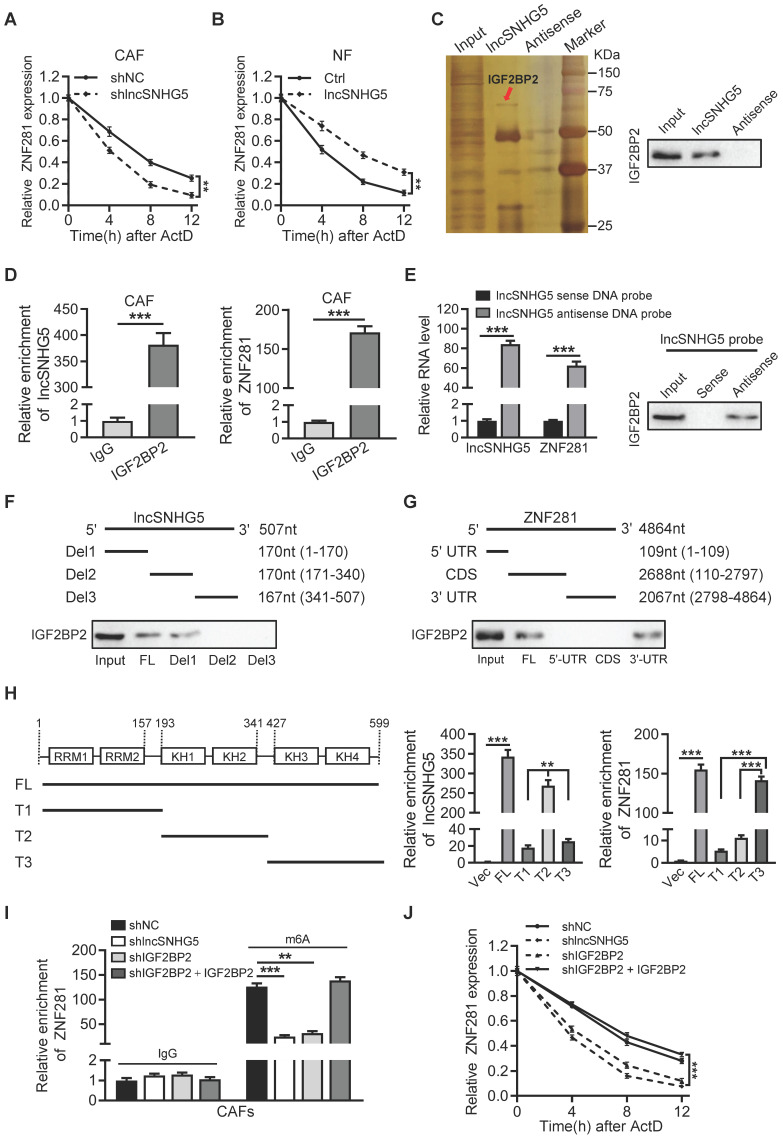
** lncSNHG5 binding with the m6A reader IGF2BP2 enhances the mRNA stability of ZNF281. (A-B)** qRT-PCR was used to determine ZNF281 mRNA levels in lncSNHG5-knockdown CAFs (A), lncSNHG5-overexpressing NFs (B) and their control cells following actinomycin D treatment at the designated time. **(C)** RNA pull-down was conducted with antisense and sense lncSNHG5 in CAF lysates, followed by silver staining and western blotting. An obviously changed band of IGF2BP2 is shown. **(D)** RIP assay for CAF lysates was performed using anti-IGF2BP2 and anti-IgG, and the enrichment of lncSNHG5 and ZNF281 in the RIP precipitates was determined by qRT-PCR. **(E)** RNA pull-down was performed with antisense or sense lncSNHG5 biotinylated DNA probe in CAF lysates, and the RNA levels of lncSNHG5 and ZNF281 and IGF2BP2 proteins in the pull-down precipitates were measured by qRT-PCR and western blotting, respectively. **(F, G)** Western blotting to examine IGF2BP2 in the pull-down precipitates with biotinylated full-length or segmented lncSNHG5 (F) or ZNF281 (G) probes in CAFs. **(H)** The lncSNHG5 and ZNF281 binding domains in IGF2BP2 were identified using full-length or truncated IGF2BP2 by RIP-qPCR. **(I)** The m6A-modified ZNF281 mRNA levels in the indicated CAF lysates were determined using a gene-specific m6A qPCR assay. **(J)** qRT-PCR to verify the relative mRNA expression of ZNF281 in the indicated CAFs treated with actinomycin. Data are presented as the mean ± SD (**P < 0.01, ***P < 0.001).

We then asked how lncSNHG5 increases ZNF281 expression. First, we tested the impact of lncSNHG5 on the transcriptional regulation of ZNF281 expression. A luciferase reporter assay showed that lncSNHG5 knockdown in CAFs or ectopic lncSNHG5 in NFs had no impact on the promoter activity of ZNF281 (Figure S4A-B). Based on the cytoplasmic distribution of lncSNHG5 (Figure 1B-C), we hypothesized that lncSNHG5 might serve as a scaffold to regulate ZNF281 mRNA stability at the posttranscriptional level, as most cytoplasmic lncRNAs do 34, 35. Indeed, when lncSNHG5-knockdown CAFs or ectopic lncSNHG5-overexpressing NFs were treated with actinomycin D, the half-life of ZNF281 mRNA dramatically decreased in lncSNHG5-knockdown CAFs in a time-dose-dependent pattern, whereas it increased in lncSNHG5-overexpressing NFs (Figure 4A-B), suggesting that lncSNHG5 plays a role in ZNF281 mRNA stability at the posttranscriptional level. To identify the potential lncSNHG5-binding proteins, we performed RNA pulldown followed by silver staining and mass spectrometry analysis. IGF2BP2 was significantly enriched in the precipitates with synthesized sense lncSNHG5 compared to antisense lncSNHG5 (Figure 4C). RNA immunoprecipitation (RIP) assays were subsequently performed using an antibody against IGF2BP2, and qRT-PCR was used to evaluate the RNA levels of lncSNHG5 and ZNF281 in the IGF2BP2 immunoprecipitates. As expected, lncSNHG5 and ZNF281 mRNA were notably enriched in the IGF2BP2 immunoprecipitates (Figure 4D). To further investigate whether IGF2BP2 could bridge lncSNHG5 and ZNF281 mRNA, we conducted a pull-down assay with a biotinylated antisense DNA probe against lncSNHG5 followed by western blotting to detect IGF2BP2, and qRT-PCR to test ZNF281 mRNA in the precipitates. The data confirmed that ZNF281 mRNA and IGF2BP2 were in the precipitates (Figure 4E), suggesting that IGF2BP2 acts as a mediator for the interaction between lncSNHG5 and ZNF281 mRNA. Next, we asked which fragment of lncSNHG5 and ZNF281 is necessary for interacting with IGF2BP2, and performed deletion-mapping and RNA pull-down assays. We found that the lncSNHG5 fragment Del1 (1-170 nt) and ZNF281 fragment 3'-UTR could bind with IGF2BP2 (Figure 4F-G), which has four K homology (KH) domains and two RNA recognition motifs (RRMs; Figure S4C). Furthermore, we observed that the KH1/2 domain (T2) of IGF2BP2 was essential for lncSNHG5 binding and that the KH3/4 domain (T3) was associated with ZNF281 (Figure 4H), suggesting a ternary complex among lncSNHG5, IGF2BP2 and ZNF281 3′-UTR.

Next, we asked whether the lncSNHG5-mediated recruitment of IGF2BP2 is necessary for ZNF281 mRNA stability. IGF2BP2 can function as an m6A reader for m6A-modified mRNAs and increase mRNA stability 36. Knockdown of lncSNHG5 had less impact on the protein level of IGF2BP2 in CAFs (Figure S4D). Loss of lncSNHG5 or IGF2BP2 in CAFs (Figure S4E) notably decreased lncSNHG5, IGF2BP2 and ZNF281 mRNA levels in the pull-down precipitates, and restoration of IGF2BP2 in IGF2BP2-knockdown CAFs recovered the levels of lncSNHG5, IGF2BP2 and ZNF281 mRNA in the precipitates (Figure S4F-H), suggesting that lncSNHG5-recruited IGF2BP2 is necessary for the formation of the ternary complex among lncSNHG5, IGF2BP2 and ZNF281. In addition, by conducting gene-specific m6A assays, we detected less m6A-modified ZNF281 mRNA in the precipitates of lncSNHG5-knockdown CAFs or IGF2BP2-knockdown CAFs, which were restored by ectopic IGF2BP2 (Figure 4I). Accordingly, we observed that ZNF281 mRNA stability (Figure 4J) and mRNA and protein levels of ZNF281 (Figure S4I-J) were significantly decreased in lncSNHG5- or IGF2BP2-knockdown CAFs, and the decreased half-life of mRNA stability and mRNA and protein levels of ZNF281 caused by loss of lncSNHG5 or IGF2BP2 could be partially rescued by ectopic IGF2BP2 in CAFs (Figure 4J, Figure S4I-J). Taken together, these data demonstrate that lncSNHG5-mediated IGF2BP2 recruitment facilitates m^6^A methylation in the 3′-UTR of ZNF281 to promote ZNF281 mRNA stability and ZNF281 protein levels.

### ZNF281 transcriptionally regulates CCL2 and CCL5 expression in CAFs

**Figure 5:**
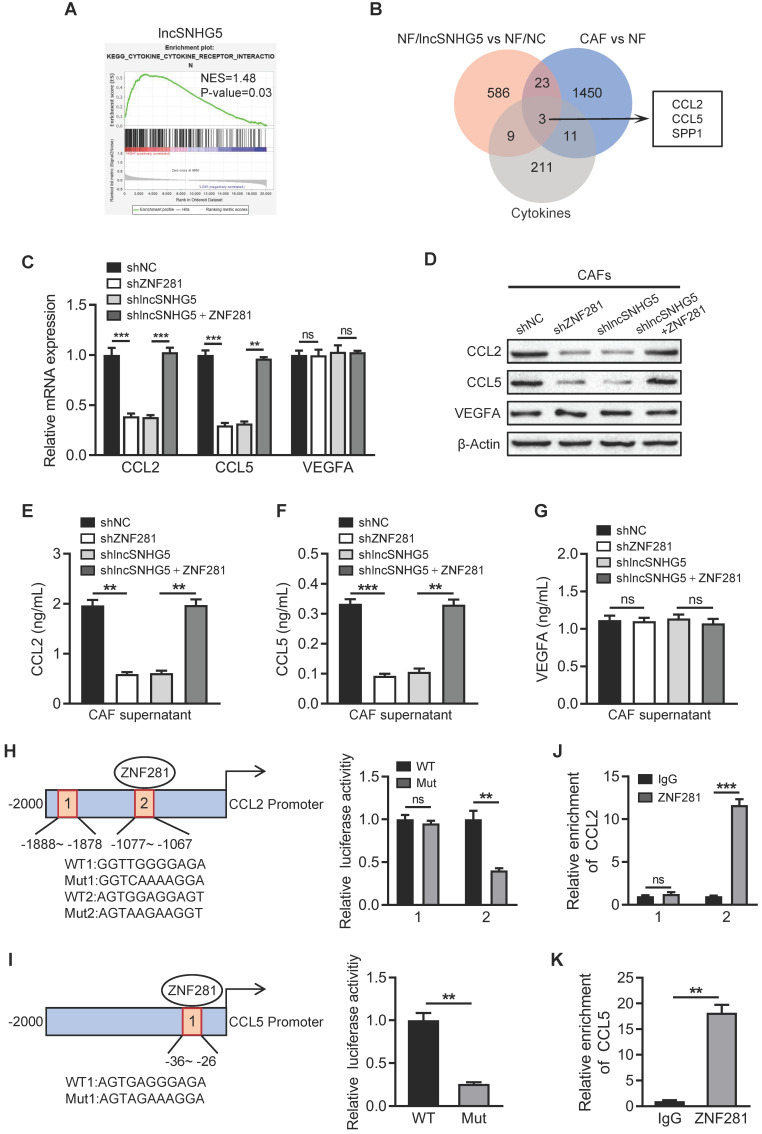
** ZNF281 transcriptionally regulates CCL2 and CCL5 expression in CAFs. (A)** Gene set enrichment analysis (GSEA) of lncSNHG5 in the KEGG pathway using TCGA data. **(B)** Venn diagram showing the cytokines as ZNF281 putative targets in CAFs. **(C-D)** qRT-PCR and western blotting were used to test the mRNA (C) and protein (D) expression levels of CCL2, CCL5 and VEGFA in CAFs/shNC, CAFs/shZNF281, CAFs/sh lncSNHG5 and CAFs/sh lncSNHG5/ZNF281. **(E-G)** ELISA was used to detect CCL2 (E), CCL5 (F) and VEGFA (G) protein levels in supernatant from the indicated engineered CAFs. **(H-I)** Schematic diagram depicting the predicted binding sites and sequences of ZNF281 in the CCL2 (H) and CCL5 (I) promoters. The relative luciferase activity was detected in CAFs transfected with wild-type (WT) or mutant luciferase reporter plasmids of CCL2 or CCL5. **(J-K)** ChIP-qPCR assay to determine ZNF281 binding to the CCL2 (J) and CCL5 (K) promoters using an anti-ZNF281 antibody. IgG was used as a negative control. Data represent the mean ± SD (ns: no significance, **P < 0.01, ***P < 0.001).

Next, we asked how lncSNHG5-mediated ZNF281 is involved in angiogenesis and vascular permeability. Using GSEA in the TCGA dataset, we found that lncSNHG5 can positively regulate cytokine-related biological processes (Figure 5A), which is consistent with the data mentioned above (Figure S1G). To identify the target cytokines of ZNF281 in breast CAFs, we compared the top 30 increased and decreased cytokines potentially regulated by ZNF281 (Figure S5A) with the upregulated cytokines acquired by microarray screen in both NFs/lncSNHG5 and primary CAFs, and found that CCL2, CCL5 and SPP1 were candidate targets of ZNF281 (Figure 5B). Furthermore, we confirmed that ectopic ZNF281 in NFs markedly upregulated stromal CCL2 and CCL5 expression and enhanced their secretion in the supernatant but had no effect on VEGFA (a known pro-angiogenic factor) levels (Figure S5B-F). Similarly, knockdown of ZNF281 or lncSNHG5 in CAFs significantly decreased CCL2 and CCL5 expression and reduced their secretion, and the loss of lncSNHG5 leading to decreased CCL2 and CCL5 was restored by ectopic ZNF281 in CAFs (Figure 5C-G). However, the VEGFA levels were not impacted by ZNF281 or lncSNHG5 in CAFs. After evaluating CCL2 and CCL5 expression in 30 pairs of CAFs and NFs by qRT‒PCR and their secreted proteins in the supernatant by ELISA, CCL2 and CCL5 were clearly higher in primary CAFs than in NFs (Figure S5G-H). In addition, the potential ZNF281-binding sites on the CCL2 and CCL5 promoters were analyzed by bioinformatics prediction (http://jaspar.genereg.net), and a luciferase reporter assay verified that ZNF281 can promote the transcriptional activity of the wild-type CCL2 and CCL5 promoters but not the mutant CCL2 and CCL5 promoters (Figure 5H-I). Chromatin immunoprecipitation (ChIP) assays further verified that ZNF281 binds to the CCL2 promoter region (-1077 to -1067 nt) and CCL5 promoter region (-36 to -26 nt) (Figure 5J-K). Taken together, these data support the idea that ZNF281 can transcriptionally regulate CCL2 and CCL5 expression.

### Breast CAF-derived CCL2 and CCL5 activate P38 MAPK signaling in endothelial cells

**Figure 6:**
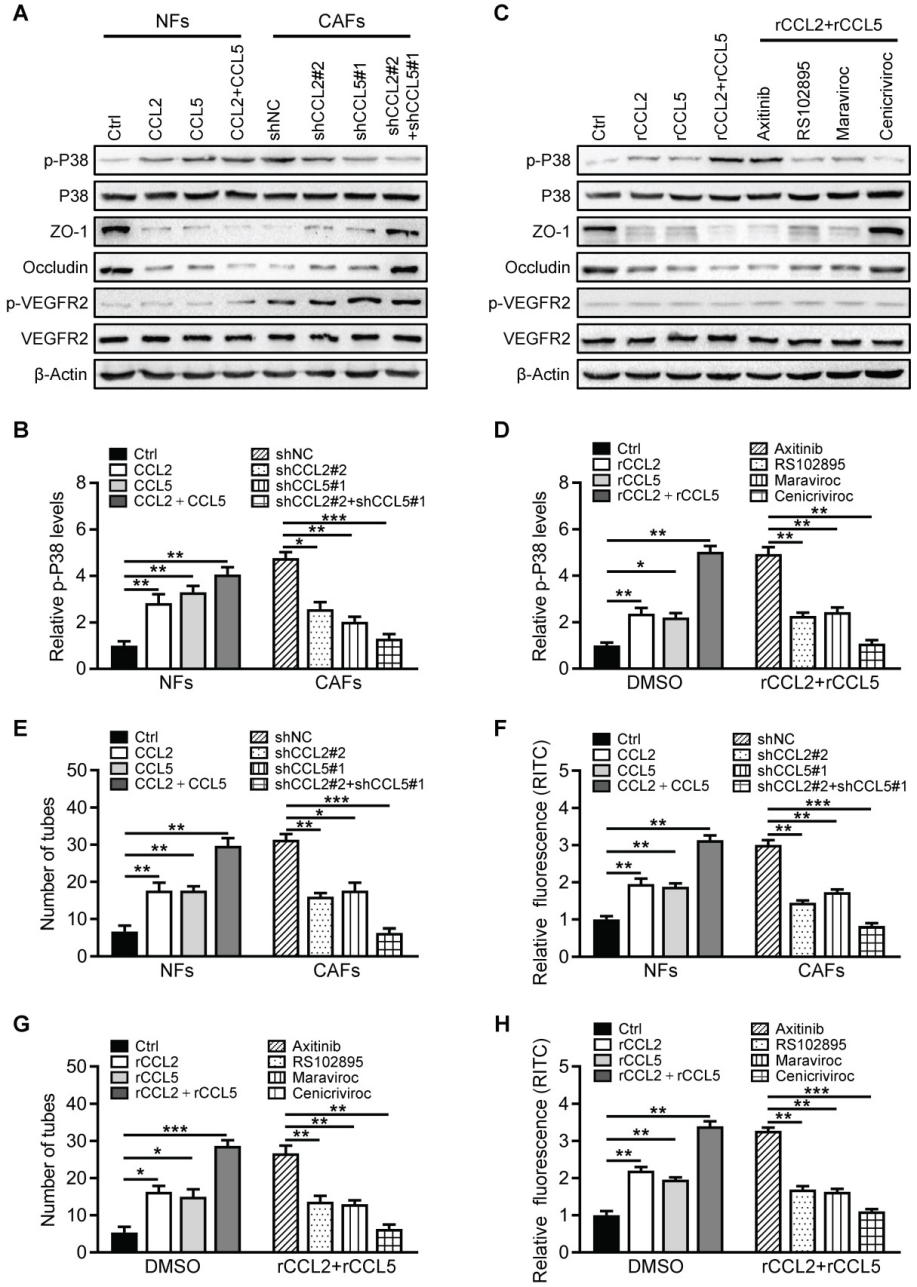
** Breast CAF-derived CCL2 and CCL5 activate P38 MAPK signaling in endothelial cells. (A-B)** Western blotting was used to analyze p-P38, P38, p-VEGFR2, VEGFR2, ZO-1, and Occludin levels in HUVECs incubated with CM from NF/Ctrl, CCL2, CCL5, CCL2/CCL5 or CAF/shNC, shCCL2, shCCL5, shCCL2/shCCL5. The pixel intensity of p-P38 was normalized to that of β-Actin for the quantification of p-P38 levels (B). **(C-D)** Western blotting was used to analyze the levels of p-P38, P38, p-VEGFR2, VEGFR2, ZO-1 and Occludin in HUVECs incubated with FBS-free medium with DMSO, rCCL2, rCCL5, rCCL2 and rCCL5 or rCCL2 and rCCL5 combined with axitinib, RS102895, maraviroc, or cenicriviroc. Levels of p-P38 (D) were quantified by normalizing its relative pixel intensity to β-Actin. **(E-F)** Effects of CM from NF/Ctrl, CCL2, CCL5, CCL2/CCL5 or CAF/shNC, shCCL2, shCCL5, shCCL2/shCCL5 on tube formation (E) and permeability (F) of HUVECs. **(G-H)** Effects of rCCL2, rCCL5, rCCL2 and rCCL5 or rCCL2 and rCCL5 combined with axitinib, RS102895, maraviroc, or cenicriviroc on tube formation (G) and permeability (H) of HUVECs. Data are presented as the mean ± SD (*P < 0.05, **P < 0.01, ***P < 0.001).

To investigate the functional role of CCL2 and CCL5 on endothelial cells, the potential downstream signaling pathways (PI3K/AKT, JAK/STAT, p38 MAPK) activated by CCL2 and CCL5 were assessed in HUVECs. Knockdown of lncSNHG5 or ZNF281 in CAFs did not affect the activation of STAT3, AKT or VEGFR2 in HUVECs. However, loss of lncSNHG5 or ZNF281 in CAFs clearly led to a reduction in phosphorylated P38 (p-P38) in HUVECs, which was rescued by ectopic ZNF281 in lncSNHG5-knockdown CAFs (Figure S6A), indicating that the lncSNHG5-ZNF281-CCL2/5 signaling axis activates P38 MAPK signaling in HUVECs. Knockdown of CCL2 and CCL5 in CAFs (Figure S6B) resulted in reduced secretion of CCL2 and CCL5 by CAFs (Figure S6D-E, right panels) and decreased p-P38 and increased ZO-1 and Occludin in HUVECs (Figure 6A-B and Figure S6F, right panels), as well as overexpression of ectopic CCL2 and CCL5 in NFs (Figure S6C), which led to increased CCL2 and CCL5 in the supernatant (Figure S6D-E, left panels), dramatically increased p-P38 levels and reduced ZO-1 and Occludin levels in HUVECs but had no effect on VEGFR activity (Figure 6A-B and Figure S6F, left panels). Furthermore, treating HUVECs with recombinant human CCL2 (rCCL2) and/or CCL5 (rCCL5) notably increased p-P38 levels and decreased ZO-1 and Occludin expression (Figure 6C-D left panels, and Figure S6G), whereas rCCL2- and rCCL5-induced changes were partially reversed by RS102895 (CCR2 inhibitor) and maraviroc (CCR5 inhibitor) and completely reversed by cenicriviroc (CCR2 and CCR5 inhibitor) but not by axitinib (VEGFR inhibitor) (Figure 6C-D right panels, and Figure S6G). Correspondingly, CM derived from CCL2- and CCL5-overexpressing stromal fibroblasts significantly facilitated tube formation and endothelial permeability of HUVECs, while CM from CCL2- and CCL5-knockdown CAFs impeded the tube formation and endothelial permeability of HUVECs (Figure 6E-F, and Figure S7A). Treatment of HUVECs with RS102895, maraviroc or cenicriviroc clearly inhibited rCCL2- and rCCL5-stimulated tube formation and vascular permeability, and cenicriviroc had the most dramatic effect (Figure 6G-H, and Figure S7B). Furthermore, inhibition of P38 MAPK signaling with SB203580 markedly abolished rCCL2- and rCCL5-induced tubule formation and vascular permeability and increased ZO-1 and Occludin levels in HUVECs (Figure S7C-E). Taken together, these data demonstrate that the lncSNHG5-ZNF281-CCL2/5 signaling axis promotes angiogenesis and vascular permeability by activating P38 MAPK signaling in HUVECs.

### LncSNHG5/ZNF281 signaling-mediated CCL2 and CCL5 secretion contributes to premetastatic niche formation by promoting angiogenesis and vascular permeability

**Figure 7:**
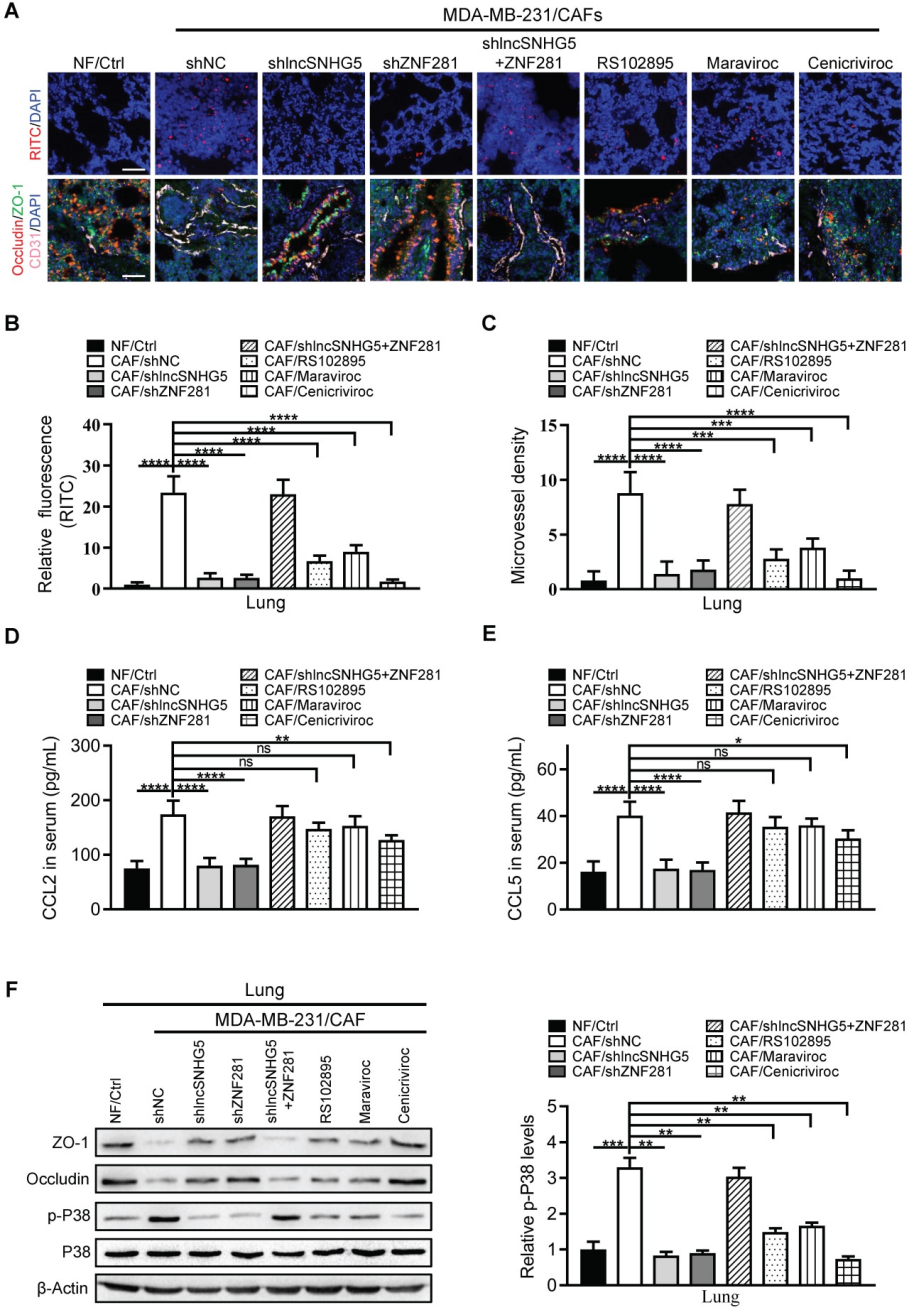
** The lncSNHG5/ZNF281 signaling axis increases CCL2 and CCL5 secretion to promote angiogenesis and vascular permeability to induce premetastatic niche formation. (A)** MDA-MB-231 cells were mixed with NFs/Ctrl, CAFs/shNC, CAFs/shlncSNHG5, CAFs/shZNF281, CAFs/shlncSNHG5/ZNF281, CAFs/RS102895, CAFs/Maraviroc or CAFs/Cenicriviroc and orthotopically inoculated into nude mice for 2 weeks. The mice were injected with rhodamine-dextran before lung metastasis, and the vascular permeability of mouse lungs in each group is shown in the upper panel (scale bar, 100 µm). Representative images of fluorescent staining of ZO-1 (green), Occludin (red) and CD31 (pink) in lung tissue are shown in the lower panel (scale bar, 100 µm). **(B)** The fluorescence levels of rhodamine-dextran in mouse lungs were calculated using ImageJ software and normalized to DAPI levels. **(C)** The microvessel density in mouse lungs was quantified by counting the microvessel numbers under the microscope. **(D-E)** The serum CCL2 and CCL5 levels in each group of mice were detected by ELISA at 2 weeks after injection. **(F)** The protein changes in ZO-1, Occludin and phosphorylated or total p38 in mouse lung tissues before metastasis were assessed by western blotting (left panel). Levels of p-P38 were quantified as pixel intensity compared with β-actin (right panel). Data are presented as the mean ± SD (**P < 0.01, ***P < 0.001. ****P < 0.0001).

To further evaluate whether the lncSNHG5-ZNF281-CCL2/CCL5 signaling axis contributes to PMN formation, we established mouse models and evaluated PMN formation via angiogenesis and vascular permeability as described above. Indeed, we observed that the permeability and MVD of pulmonary capillaries were significantly increased in the tumor-bearing mice injected with MDA-MB-231 and CAFs (MDA-MB-231/CAFs shNC) before lung metastasis, and the expressions of ZO-1 and Occludin were decreased. By contrast, the lung capillary permeability and MVD were markedly decreased in the tumor-bearing mice injected with MDA-MB-231 and NFs (MDA-MB-231/NFs Ctrl) or CAFs with shRNA against lncSNHG5 and ZNF281 (CAFs/sh lncSNHG5, CAFs/shZNF281), or CAFs with inhibitors RS102895 or Maraviroc or Cenicriviroc (CAFs/RS102895, CAFs/Maraviroc, CAFs/Cenicriviroc), and the ZO-1 and Occludin levels were increased in these groups. lncSNHG5 knockdown induced a decrease of lung vascular permeability and angiogenesis, and the increase of ZO-1 and Occludin was rescued by overexpression of ectopic ZNF281 in CAFs (Figure 7A-C). Correspondingly, the serum CCL2 and CCL5 levels were highest in the mice injected with MDA-MB-231/CAFs shNC and MDA-MB-231/CAFs sh lncSNHG5/ZNF281, and decreased serum CCL2 and CCL5 levels were observed in tumor-bearing mice injected with MDA-MB-231/CAFs shRNAs against lncSNHG5 and ZNF281 (CAFs/sh lncSNHG5, CAFs/shZNF281; Figure 7D-E). Consistent with the changes in serum CCL2 and CCL5 in tumor-bearing mice, there were corresponding changes in ZO-1, Occludin and phosphorylated p38 protein levels in these premetastatic mouse lungs. In addition, after injection of RS102895 or maraviroc or cenicriviroc to block the binding of CCL2/CCR2 or/and CCL5/CCR5, the levels of phosphorylated p38 were decreased, and the levels of ZO-1 and Occludin were increased (Figure 7F). These data illustrate that the lncSNHG5-ZNF281 axis induction of enhanced CCL2 and CCL5 in CAFs facilitates angiogenesis and permeability of pulmonary capillaries to promote lung premetastatic niche formation *in vivo*.

### The lncSNHG5-ZNF281-CCL2/CCL5 signaling axis facilitates BC metastasis

**Figure 8:**
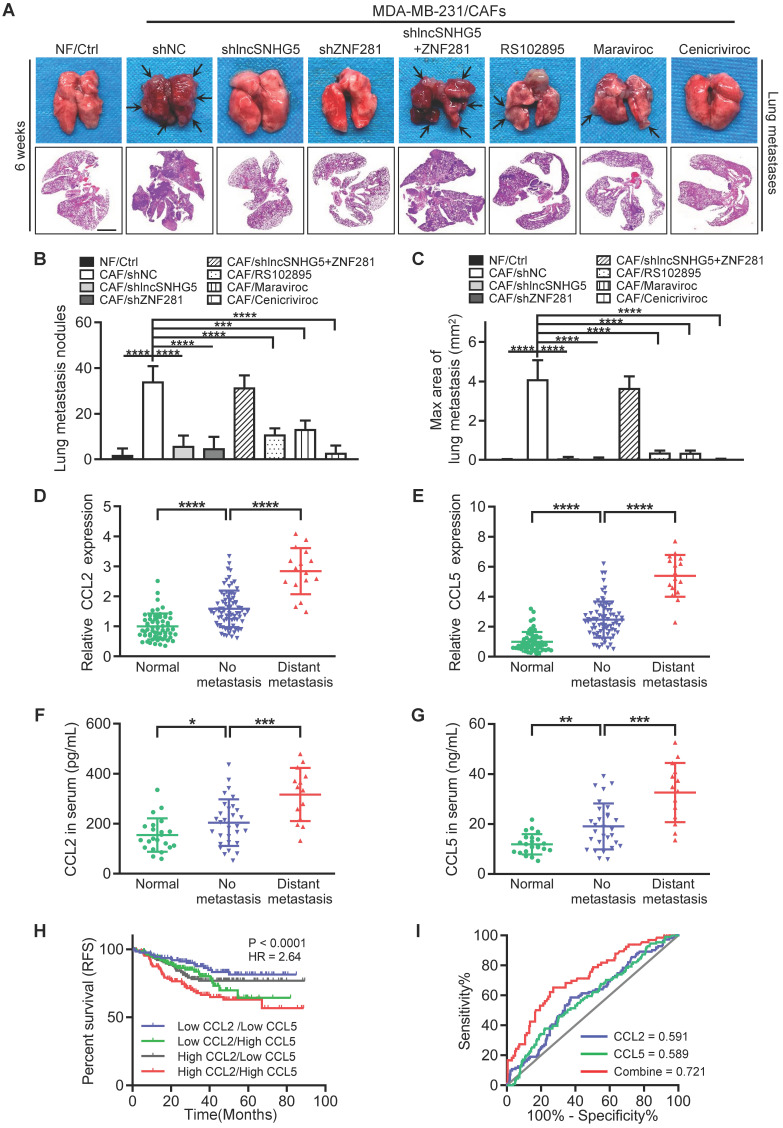
** The lncSNHG5-ZNF281-CCL2/CCL5 signaling axis facilitates BC metastasis. (A)** A mixture of MDA-MB-231 cells and NFs/Ctrl, CAFs/shNC, CAFs/shlncSNHG5, CAFs/shZNF281, CAFs/shlncSNHG5/ZNF281, CAFs/RS102895, CAFs/Maraviroc or CAFs/Cenicriviroc was orthotopically injected into nude mice for 6 weeks. Representative images of pulmonary metastases in the lung surface (upper panel) and lung section (scale bar, 200 µm) by H&E staining (lower panel) are shown. **(B)** The number of lung metastatic nodules in each group of mice was counted under a microscope. **(C)** The maximal area of lung metastasis in each group was quantified using SlideViewer software. **(D-E)** qRT-PCR was used to detect the mRNA expression of CCL2 and CCL5 in human breast tumors with (16 cases) or without (76 cases) metastasis and adjacent normal tissues (60 cases). **(F-G)** ELISA was used to determine CCL2 and CCL5 proteins in healthy donors (22 cases) and breast cancer patients with (14 cases) or without (30 cases) metastasis. **(H)** Kaplan-Meier survival analysis of relapse-free survival based on the combined low or high levels of CCL2 and CCL5 using the GSE25066 dataset. **(I)** The diagnostic value of CCL2 and CCL5 for poor BC prognosis was evaluated using ROC curves. Data are presented as the mean ± SD (*P < 0.05, **P < 0.01, ***P < 0.001. ****P < 0.0001).

It has been previously reported that the establishment of a premetastatic niche is critical for the colonization of tumor cells in distant metastatic organs 37. To determine whether lncSNHG5-ZNF281-CCL2/CCL5 signaling in CAFs promotes BC metastasis, we established a metastatic mouse model for 6 weeks. Compared with the tumor-bearing mice injected with MDA-MB-231 cells and CAFs, the tumor-bearing mice injected with the mixture of MDA-MB-231 cells and NFs or CAFs with shRNA against lncSNHG5 and ZNF281 (CAF/sh lncSNHG5, CAF/shZNF281) or CAFs with inhibitors RS102895 or marviroc or cenicriviroc (CAFs/RS102895, CAFs/Maraviroc, CAFs/Cenicriviroc) had fewer and smaller lung metastases, whereas overexpression of ectopic ZNF281 in the lncSNHG5-knockdown CAFs (CAFs/sh lncSNHG5/ZNF281) rescued the lung metastases (Figure 8A-C). Accordingly, the serum levels of CCL2 and CCL5 were accompanied by lncSNHG5 and ZNF281 changes in these mouse models (Figure S8A-B). Taken together, these data illustrate that the lncSNHG5-ZNF281-CCL2/CCL5 signaling axis in CAFs plays an important role in breast cancer metastasis *in vivo*.

To expand our findings of CCL2 and CCL5 regulated by lncSNHG5-ZNF281 signaling in promoting tumor metastasis, we evaluated CCL2 and CCL5 levels in tumor tissues and serum CCL2 and CCL5 of healthy donors and BC patients with or without metastasis. Our data showed that CCL2 and CCL5 were more highly expressed in BC tissues than in normal tissues and that these proteins were increased to a greater extent in tumors with distant metastasis than in those without metastasis (Figure 8D-E). Similarly, high levels of serum CCL2 and CCL5 were observed in BC patients in contrast to healthy donors, and metastatic BC patients had higher levels of serum CCL2 and CCL5 than those without metastasis (Figure 8F-G). Analysis of the TCGA and GSE25066 datasets showed that high levels of CCL2 or CCL5 were closely related to distant metastasis (Figure S8C-D) and poor relapse-free survival (Figure S8E-F). Interestingly, when both CCL2 and CCL5 were highly expressed, patients had the worst prognosis (Figure 8H). In addition, the high levels of CCL2 and CCL5 were a good diagnostic index of poor prognosis for BC patients as assessed by receiver operating characteristic (ROC) curve (Figure 8I). Taken together, our work demonstrates that activated stromal lncSNHG5-ZNF281-CCL2/CCL5 signaling plays a vital prometastatic role in BC.

## Discussion

Angiogenesis and vascular permeability are crucial for PMN formation 4. The activated CAFs in the tumor microenvironment (TME) that promote tumor progression are well known. However, the molecular mechanisms underlying breast CAFs in PMN formation remain less understood. In the current study, we show that lncSNHG5 is enhanced in breast CAFs and is closely related to poor survival of BC patients. The increased lncSNHG5 in breast CAFs plays a key role in PMN formation by regulating angiogenesis and vascular permeability by recruiting IGF2BP2 to increase ZNF281 mRNA stability in an m6A-dependent pattern. Furthermore, ZNF281 in CAFs upregulated CCL2 and CCL5 rather than VEGFA expression, which stimulated angiogenesis and vascular permeability through P38 MAPK signaling in endothelial cells, consequently contributing to lung PMN formation in tumor metastasis.

The functions of lncRNAs depend on their subcellular localization. It is well established that nuclear lncRNAs are involved in transcriptional regulation, chromatin modification and RNA processing, while cytoplasmic lncRNAs are involved in maintaining the stability and translation of mRNA and affect cell signaling cascades 38. However, many lncRNAs remain unknown. lncSNHG5 was previously reported to play a role in tumors mainly through competitive binding of miRNA. For example, lncSNHG5 works as a ceRNA by binding with miR-26a-5p, miR-132-3p or miR-154-5p to increase PCNA, GSK3β and CREB5 expression to drive cell proliferation, migration and metastasis of tumor cells 39-41. Here, we show that stromal lncSNHG5 is largely localized in the cytoplasm of CAFs and interacts with IGF2BP2, which functions as a reader of N6-methyladenosine (m6A) to recognize m6A sites through the KH domain 36. Several studies have shown that IGF2BP2, as an RNA-binding protein, carries out a binding role with specific mRNAs to increase their stability. For instance, IGF2BP2 enhances DANCR stability, functioning as an m6A reader for m6A-modified DANCR mRNA in the maintenance of pancreatic cancer stemness 42. Moreover, LINC00460 was found to interact with IGF2BP2 in promoting the proliferation and metastasis of colorectal cancer by increasing the stability of HMGA1 mRNA via an m6A-mediated modification 43. Here, we show that lncSNHG5-mediated recruitment of IGF2BP2 binds to the mRNA 3'-UTR of ZNF281, a transcription factor 44, and increases ZNF281 levels through m6A modification to regulate CCL2 and CCL5 transcription and secretion, leading to PMN formation. Nevertheless, the m6A modification status may also depend on the function of m6A methyltransferase or m6A demethylases 45; whether there are aberrantly expressed methylases or demethylases in CAFs that play a pivotal role in the m6A modification of ZNF281 needs further exploration.

Premetastatic niche formation is the precursor or determinant of tumor colonization and the emergence of metastases in distant organs. Previous studies have found that primary tumors can promote PMN formation by secreting factors and tumor-shed extracellular vesicles 5. It has been reported that primary tumor-released LOXL2 can promote the expression of FN, MMP9 and CXCL12 and the recruitment of bone marrow-derived cells (BMDCs) to facilitate the generation of lung PMN in hepatocellular carcinoma 46, 47. In addition, BC cell-derived exosomal miR-21 contributes to PMN formation by inducing osteoclast differentiation to promote bone metastasis 48. However, the role of primary TME stromal cells in the formation of the PMN in distant organs is less well studied. Only a few studies have shown that primary TME stromal cells may participate in PMN construction. For example, CYP4A in tumor-associated macrophages is involved in PMN formation and metastasis by regulating the expression of TGF-β, SDF-1 and VEGF 49. A recent study showed that CAF-derived EVs boost lung PMN formation in salivary adenoid cystic carcinoma 22. Here, we confirmed that primary CAFs are involved in PMN formation via angiogenesis and vascular permeability in breast cancer.

In the current study, we demonstrate that the lncSNHG5-ZNF281 signaling axis in breast CAFs plays a central role in lung PMN formation in breast cancer by regulating CCL2 and CCL5 expression. Notably, angiogenesis and vascular permeability are the key steps in PMN formation. Cancer-secreted miR-105 can destroy vascular endothelial barriers to accelerate BC metastasis by decreasing ZO-1 levels during the early premetastatic stage 7. Calcineurin-NFAT-angiopoietin-2 signaling was found to activate endothelial cells to trigger angiogenesis in early metastatic lesions 50. Moreover, VEGFA is considered a crucial factor in regulating angiogenesis and vascular permeability 51. Intriguingly, our study revealed that the lncSNHG5-ZNF281 signaling axis plays a key role in VEGF-independent angiogenesis and vascular leakage by regulating CCL2 and CCL5 expression in CAFs. This may be a reason for the limited effectiveness of antiangiogenic therapy for some solid tumors, including breast cancer. CCL2-CCR2 signaling has been shown to trigger vascular permeability and metastasis via p38 MAPK and JAK2-STAT5 signaling 52. This is in line with our findings that CAF-derived CCL2 and CCL5 bind to CCR2 and CCR5 receptors, specifically activating p38 MAPK signaling in endothelial cells, thus critically impacting PMN formation. In this study, we verified that lncSNHG5-ZNF281-mediated CCL2/CCL5 release into serum notably decreases ZO-1 and Occludin levels in endothelial cells, increases MVD and leads to activation of p-P38 in lung tissues, which promotes angiogenesis and permeability of pulmonary capillaries to facilitate PMN generation in lung tissues before the occurrence of lung metastasis. Excitingly, blocking CCL2/CCR2 and CCL5/CCR5 signaling *in vivo* with the inhibitors RS102895, maraviroc and cenicriviroc significantly inhibited angiogenesis and vascular permeability in the lung PMN, especially cenicriviroc, which inhibited both CCR2 and CCR5 and was observed to have a powerful inhibitory effect on BC metastasis in this study, indicating a new strategy for the clinical prevention and treatment of BC metastasis.

## Conclusions

Taken together, our work highlights the role of stromal lncSNHG5 in CAFs in PMN formation via ZNF281-CCL2/5-p-P38 signaling in the regulation of angiogenesis and vascular permeability, thus triggering tumor metastasis in BC. The enhanced lncSNHG5 and CCL2/CCL5 may serve as potential novel diagnostic markers, and RS102895, maraviroc or cenicriviroc, and inhibitors of CCR2 and/or CCR5, may be potential molecules to prevent lung metastasis in BC patients.

## Supplementary Material

Supplementary figures and tables.

CAF VS NF (LncRNA).

## Abbreviations

LncRNA: Long noncoding RNA
BC: Breast cancer
CAFs: Cancer-associated fibroblasts
PMN: Premetastatic niche
NFs: Normal fibroblasts
HUVECs: Human umbilical vein endothelial cells
TME: Tumor microenvironment
TCGA: The Cancer Genome Atlas
KEGG: Kyoto Encyclopedia of Genes and Genomes
GSEA: Gene Set Enrichment Analysis
CM: Conditioned medium
qRT‒PCR: Real-time quantitative polymerase chain reaction
FISH: Fluorescence *in situ* hybridization
IHC: Immunohistochemistry
WB: Western blotting
ELISA: Enzyme-linked immunosorbent assay
RIP: RNA immunoprecipitation
3'-UTR: 3'-untranslated region
RRM: RNA recognition motifs
KH: K homology
CHIP: Chromatin Immunoprecipitation
MVD: Microvessel density
ROC: Receiver operating characteristic
