# Supplementary material for: Cancer-associated fibroblasts facilitate premetastatic niche formation through lncRNA SNHG5-mediated angiogenesis and vascular permeability in breast cancer
Source: Theranostics. 2022 Oct 17;12(17):7351–70. doi: 10.7150/thno.74753 (PMC9691361; doi:10.7150/thno.74753)
Supplement: Supplementary file 1 — Supplementary figures and tables. [file thnov12p7351s1.pdf]

Supplementary Figures and legends

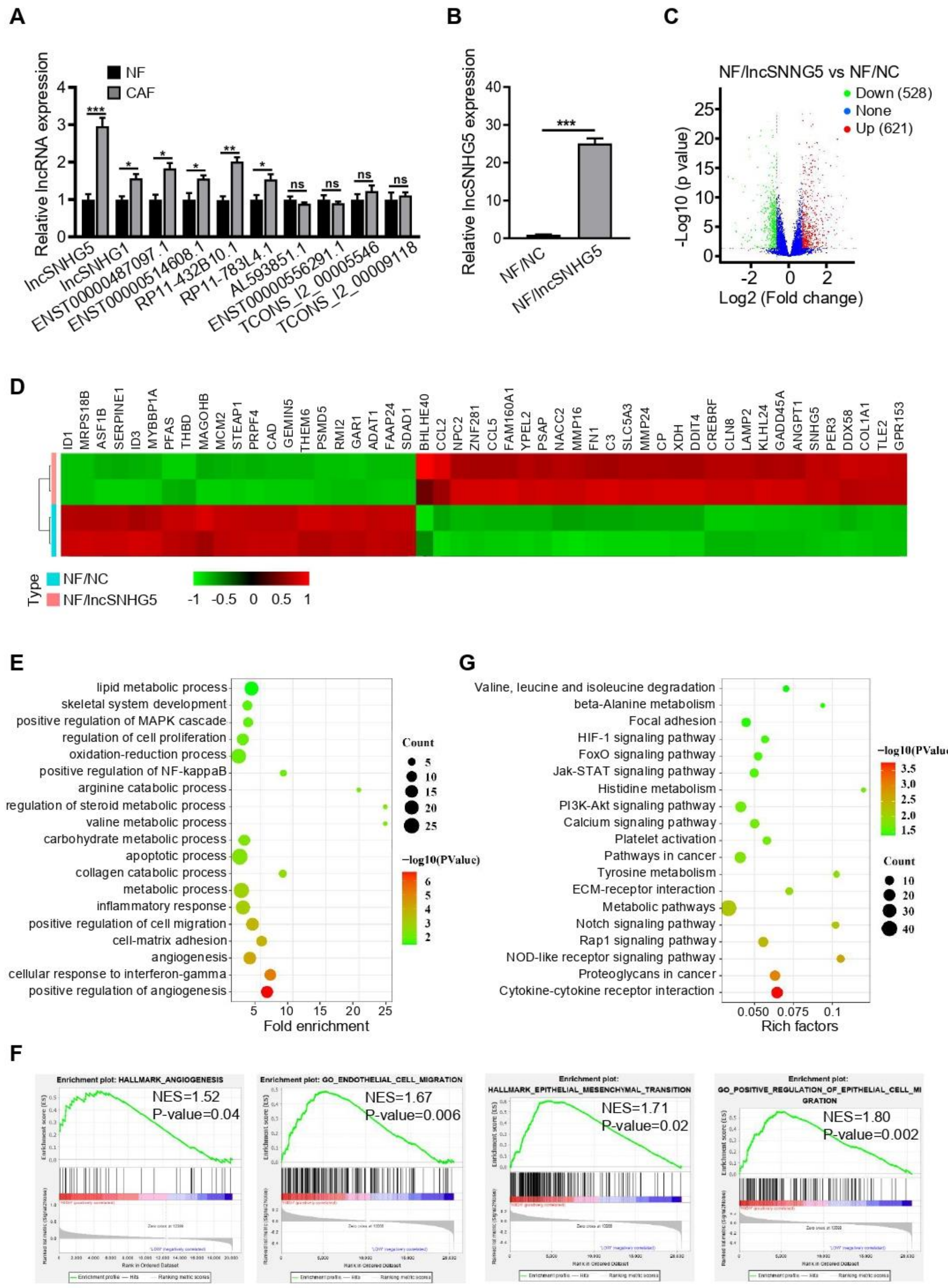

Figure S1. lncSNHG5 is closely related to angiogenesis and metastasis

(A) Validation of the aberrantly enhanced lncRNAs in the microarray. Ten of the

upregulated lncRNAs were randomly selected and confirmed using qRT–PCR in paired CAFs and NFs. (B) The overexpression efficiency of lncSNHG5 in NFs was verified using Q-PCR. (C) Volcano plot displaying differential gene expression between lncSNHG5-overexpressing NFs (NFs/lncSNHG5) and control NFs (NFs/NC). (D) The heatmap shows the top 50 dysregulated genes. (E) Gene ontology (GO) enrichment analysis enriched in upregulated genes. (F) Gene set enrichment analysis (GSEA) of lncSNHG5 showing angiogenesis and cell migration as significantly enriched in TCGA data. (G) The upregulated genes were used in KEGG pathway enrichment analysis. The data are presented as the mean ± SD (ns: no significance, *$P < 0.05$, **$P < 0.01$, ***$P < 0.001$).

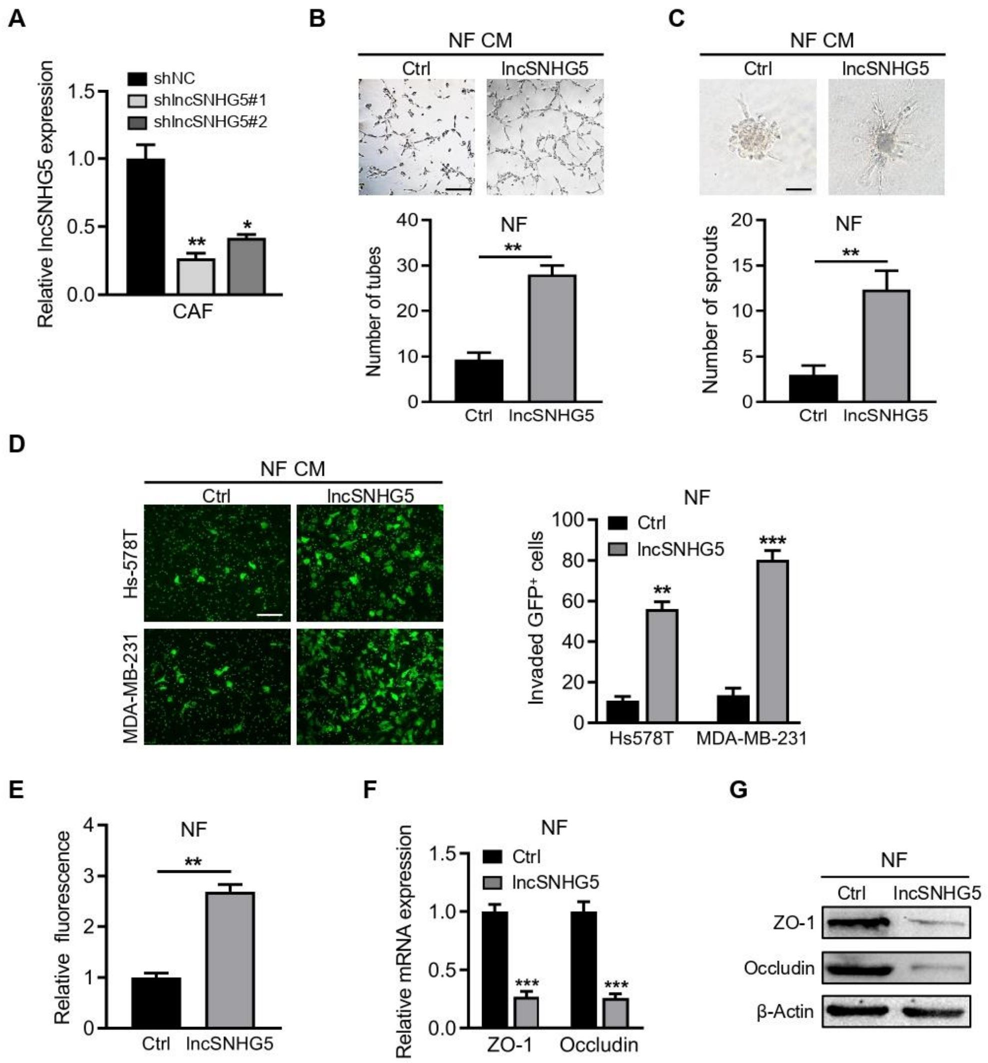

Figure S2. Overexpression of lncSNHG5 in breast NFs induces angiogenesis and vascular leakiness

(A) qRT–PCR was used to evaluate the knockdown efficiencies of lncSNHG5 in CAFs. (B, C) After treatment with CM from lncSNHG5-overexpressing NFs and control NFs, tube formation (B) or spheroid sprouting ability (C) of HUVECs was assessed by tube formation or three-dimensional sprouting assays (scale bar, 100 μm). (D, E) After treatment with CM from the indicated cells, HUVEC monolayers were constructed. Then, the permeability of HUVECs was assessed using a transwell assay based on the invasive GFP^+ MDA-MB-231 and Hs578T cells (D) (scale bar, 200 μm), and

rhodamine-dextran crossed across the endothelial cell monolayer (E). (F, G) The mRNA (F) and protein (G) levels of ZO-1 and Occludin in HUVECs treated with CM from lncSNHG5-overexpression NFs and control NFs were determined using qRT–PCR and WB. The data are presented as the mean ± SD (**$P < 0.01$, ***$P < 0.001$).

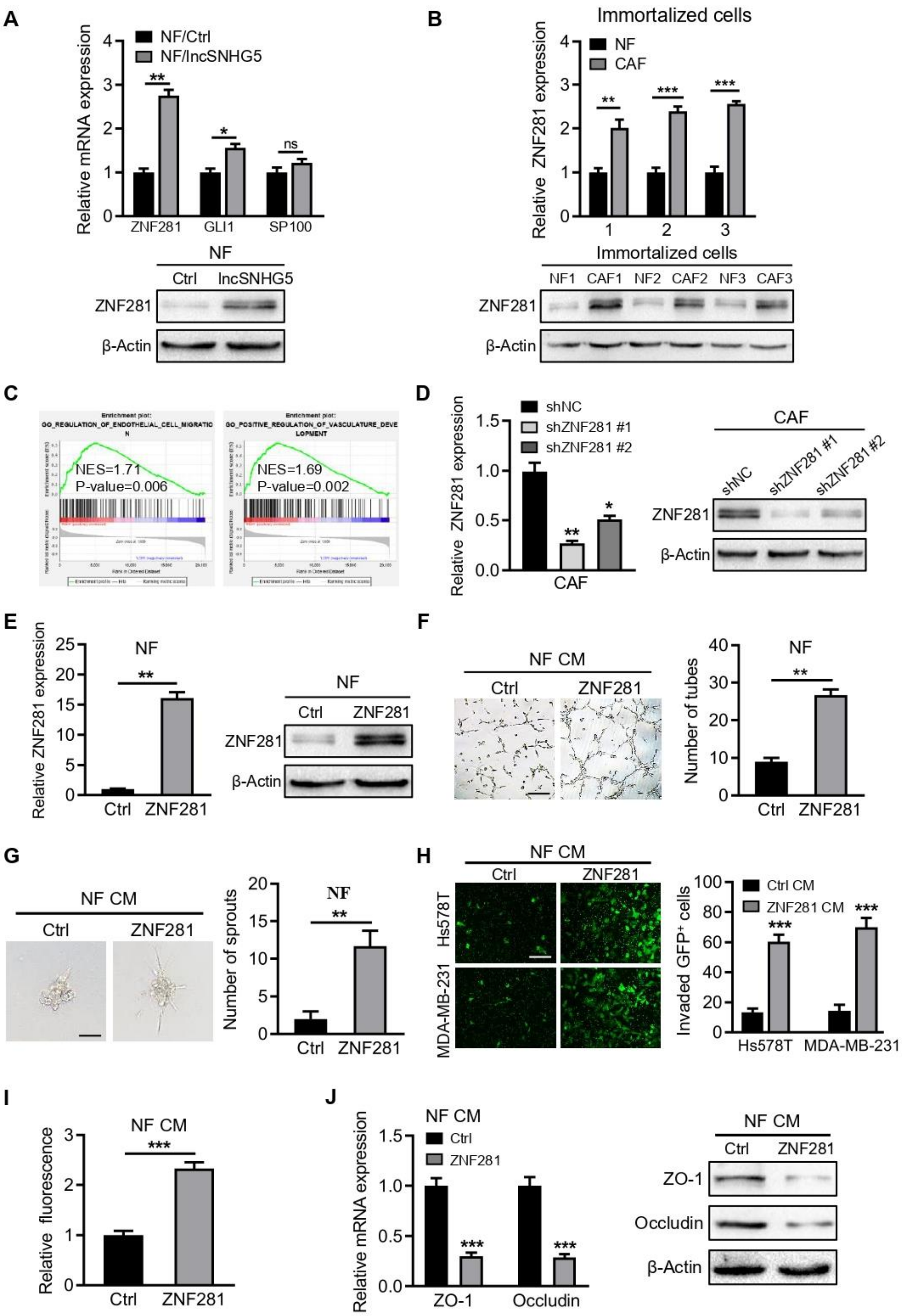

Figure S3. LncSNHG5 promotes angiogenesis and endothelial permeability by regulating ZNF281 in CAFs

(A) qRT–PCR and WB were used to test the levels of ZNF281 in lncSNHG5-overexpressing NFs and control NFs. (B) Validation of ZNF281 expression in 3

immortalized CAFs using qRT–PCR and WB. (C) GSEA of ZNF281 in biological processes using TCGA data. (D) ZNF281 knockdown efficiency in CAFs was measured by qRT–PCR and WB. (E) Validation of ZNF281 overexpression in NFs using qRT–PCR and WB. (F, G) Effect of CM derived from NF/Ctrl and NF/ZNF281 on tube formation (F) and sprouting spheroid (G) functions of HUVECs (scale bar, 100 μm). (H, I) HUVECs were cultured with CM derived from the indicated NFs to form monolayers, and then the permeability of HUVECs was assessed either by transwell assay using GFP-labeled MDA-MB-231 and Hs578T cells (H) or by rhodamine-dextran fluorescence detection (I) (Scale bar, 100 μm). (J) qRT–PCR and WB were used to measure ZO-1 and Occludin mRNA and protein levels in HUVECs cocultured with CM from ZNF281-overexpressing NFs and control NFs. The data are presented as the mean ± SD (ns: no significance, $^*P < 0.05$, $^{**}P < 0.01$, $^{***}P < 0.001$).

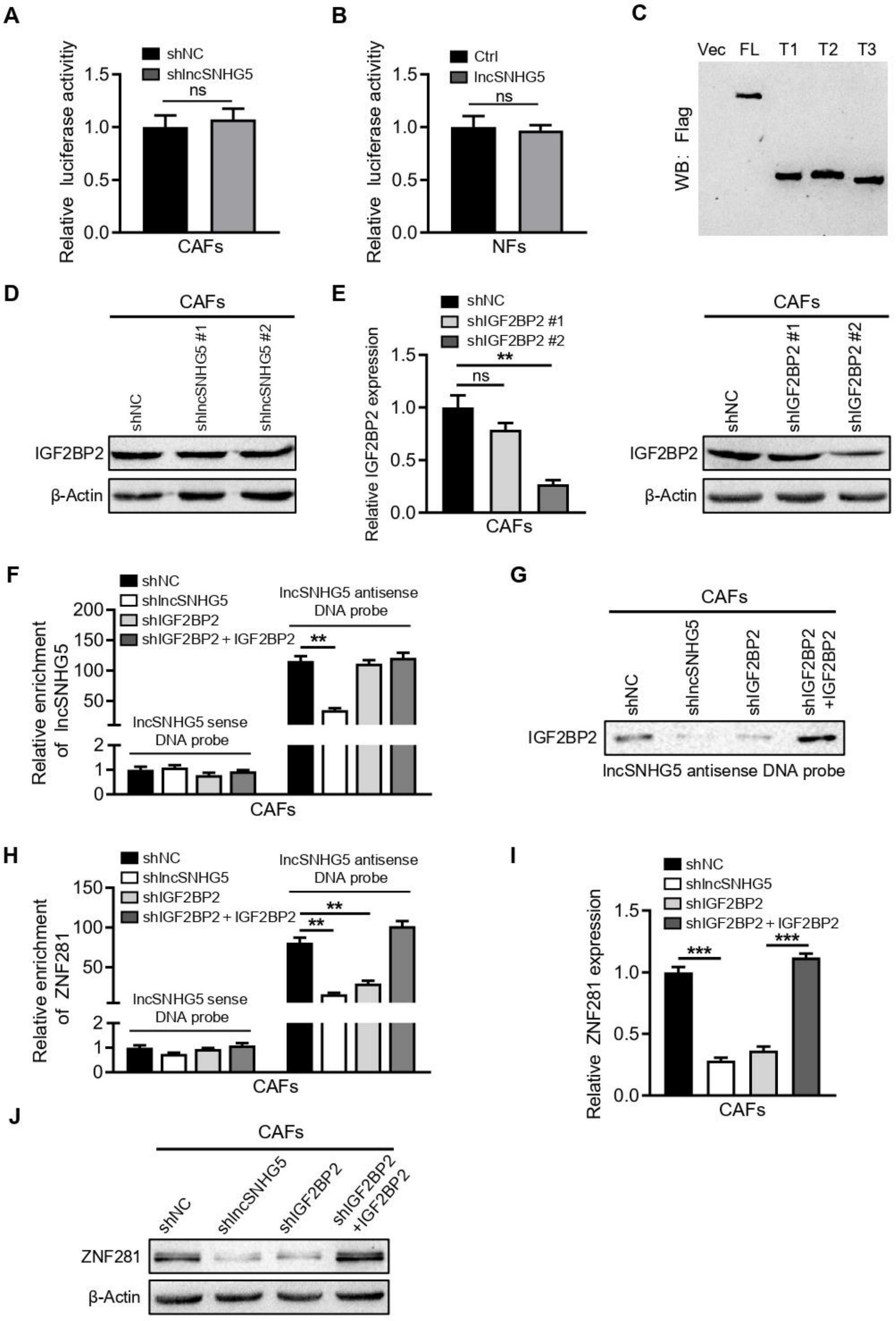

Figure S4. lncSNHG5 binding with the m6A reader IGF2BP2 enhances ZNF281 mRNA stability

(A, B) Luciferase reporter assays showed that lncSNHG5 knockdown in CAFs or ectopic lncSNHG5 overexpression in NFs had less impact on the promoter activity of

ZNF281. (C) Deletion mappings of IGF2BP2 were analyzed by western blotting. (D) WB was used to analyze IGF2BP2 protein levels in CAFs with or without sh lncSNHG5. (E) Knockdown efficiency for IGF2BP2 in CAFs was evaluated using qRT–PCR and WB. (F-H) The indicated engineered CAF cell lysates were incubated with antisense or sense biotinylated lncSNHG5 DNA probes, and an RNA pull-down assay was performed. The lncSNHG5 (F), IGF2BP2 protein (G), or ZNF281 mRNA (H) in the pull-down precipitates were detected using qRT-PCR and WB. (I, J) The mRNA (I) and protein (J) expression levels of ZNF281 in each group were validated using qRT–PCR and WB. Data are shown as the mean ± SD (ns: no significance, $^{**}P < 0.01$, $^{***}P < 0.001$).

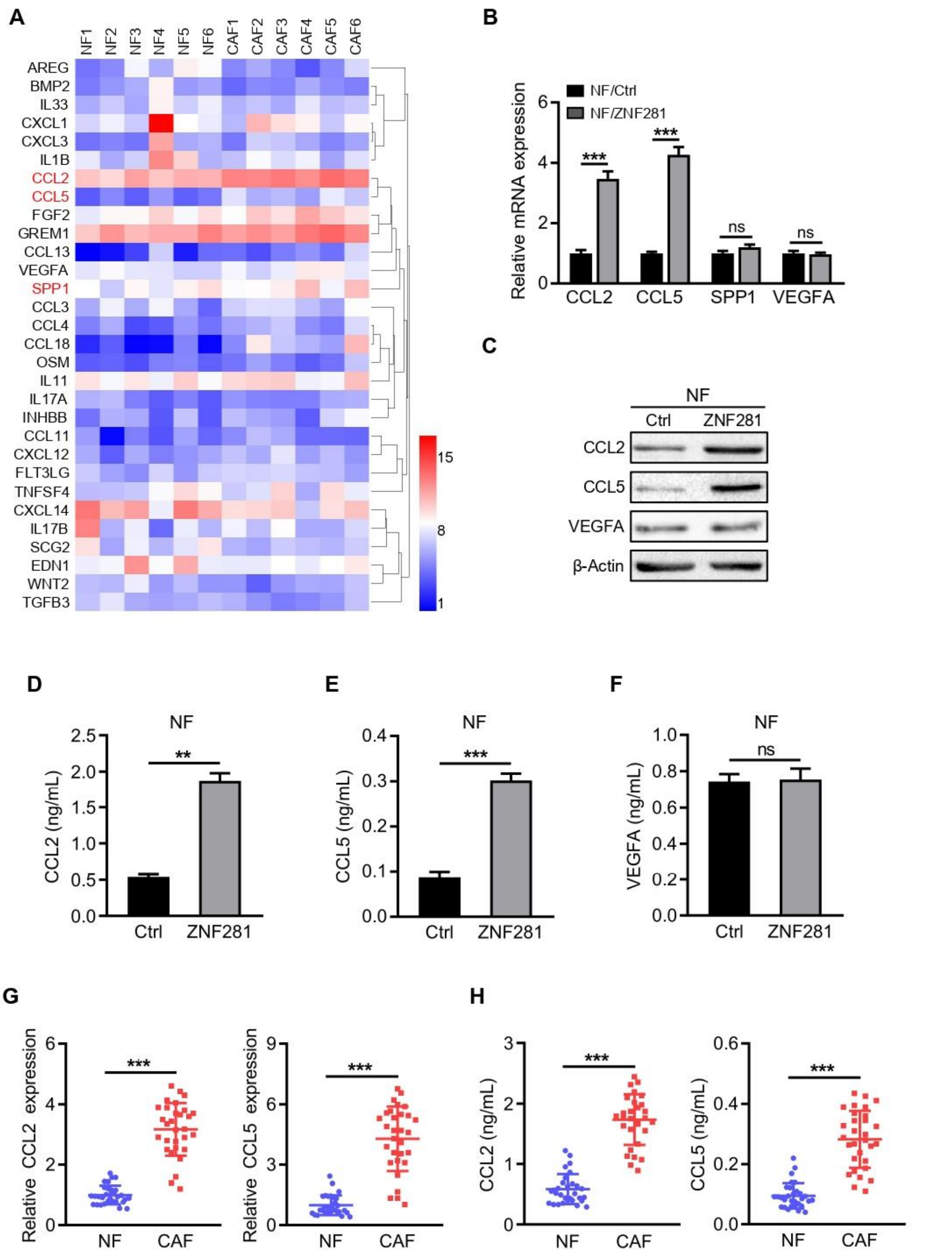

Figure S5. ZNF281 regulates CCL2 and CCL5 expression in CAFs

(A) Heatmap of the top 30 upregulated and downregulated cytokines in primary NFs and CAFs. (B-C) The levels of CCL2, CCL5, SPP1 and VEGFA were evaluated using qRT–PCR (B) and WB (C) in NFs with ectopic ZNF281 and control NFs. (D-F) ELISA

was used to determine the levels of secreted CCL2 (D), CCL5 (E) and VEGFA (F) proteins in the supernatant from ZNF281-overexpressing NFs and control NFs. (G) The levels of CCL2 and CCL5 in 30 pairs of CAFs and NFs were evaluated using qRT–PCR. (H) The secreted CCL2 and CCL5 levels in supernatant derived from 30 pairs of NFs and CAFs were examined using ELISA. Data represent the mean ± SD (ns: no significance, $^{**}P < 0.01$, $^{***}P < 0.001$).

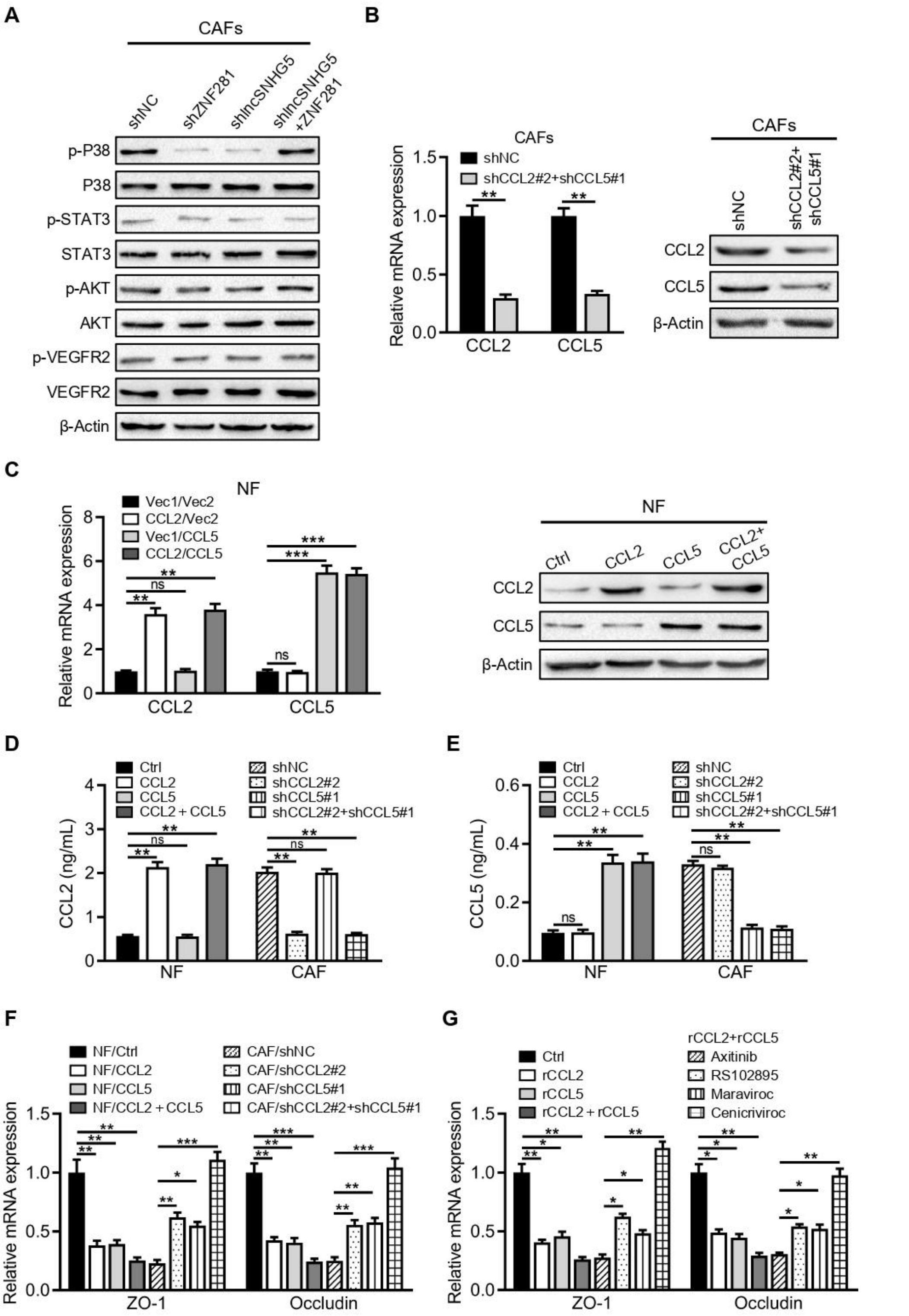

Figure S6. Validation of CCL2 and CCL5 knockdown in CAFs or overexpression in NFs

(A) Western blotting was used to determine phosphorylated or total p38, STAT3, AKT and VEGFR2 protein levels in HUVECs incubated with CM from lncSNHG5 or

ZNF281-knockdown CAFs or in lncSNHG5-knockdown CAFs with ectopic ZNF281. (B) Knockdown efficiencies of CCL2 and CCL5 in CAFs were verified using qRT–PCR and WB. (C) Validation of ectopic CCL2 and CCL5 expression in NFs using qRT–PCR and WB. (D, E) ELISA was performed to detect the secreted CCL2 and CCL5 in the above cells. (F, G) qRT–PCR was used to determine ZO-1 and Occludin levels in HUVECs treated with CM from the above groups. Data represent the mean ± SD (ns: no significance, $*P < 0.05$, $**P < 0.01$, $***P < 0.001$).

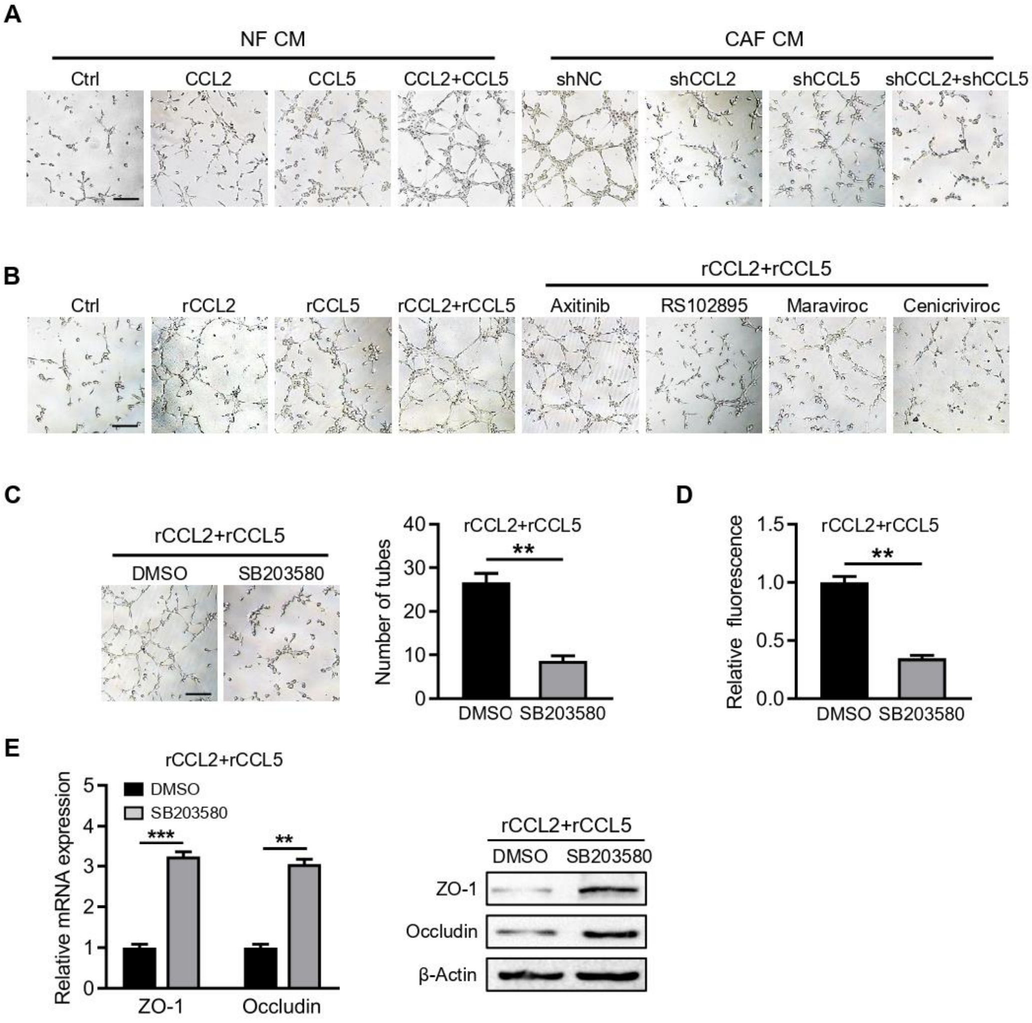

Figure S7. Breast CAF-derived CCL2 and CCL5 activate P38 MAPK signaling in endothelial cells

(A) Representative tubule formation images of HUVECs incubated with CM derived from CCL2- and CCL5-overexpressing NFs or CM from CCL2- and CCL5-knockdown CAFs. (B) Representative tube formation images of HUVECs treated with rCCL2, rCCL5, rCCL2 and rCCL5, or rCCL2 and rCCL5 combined with axitinib, RS102895, maraviroc or cenicriviroc. (C) Representative images of tube formation after SB203580 treatment with rCCL2 and rCCL5 in HUVECs. The quantification of tube formation is shown. (D) Effects of SB203580 combined with rCCL2 and rCCL5 on HUVEC monolayer permeability. (E) qRT–PCR and WB were used to analyze the levels of ZO-1 and Occludin in HUVECs treated with SB203580 and rCCL2 and rCCL5. Scale bar, 100 μm. Data represent the mean ± SD (**P < 0.01, ***P < 0.001).

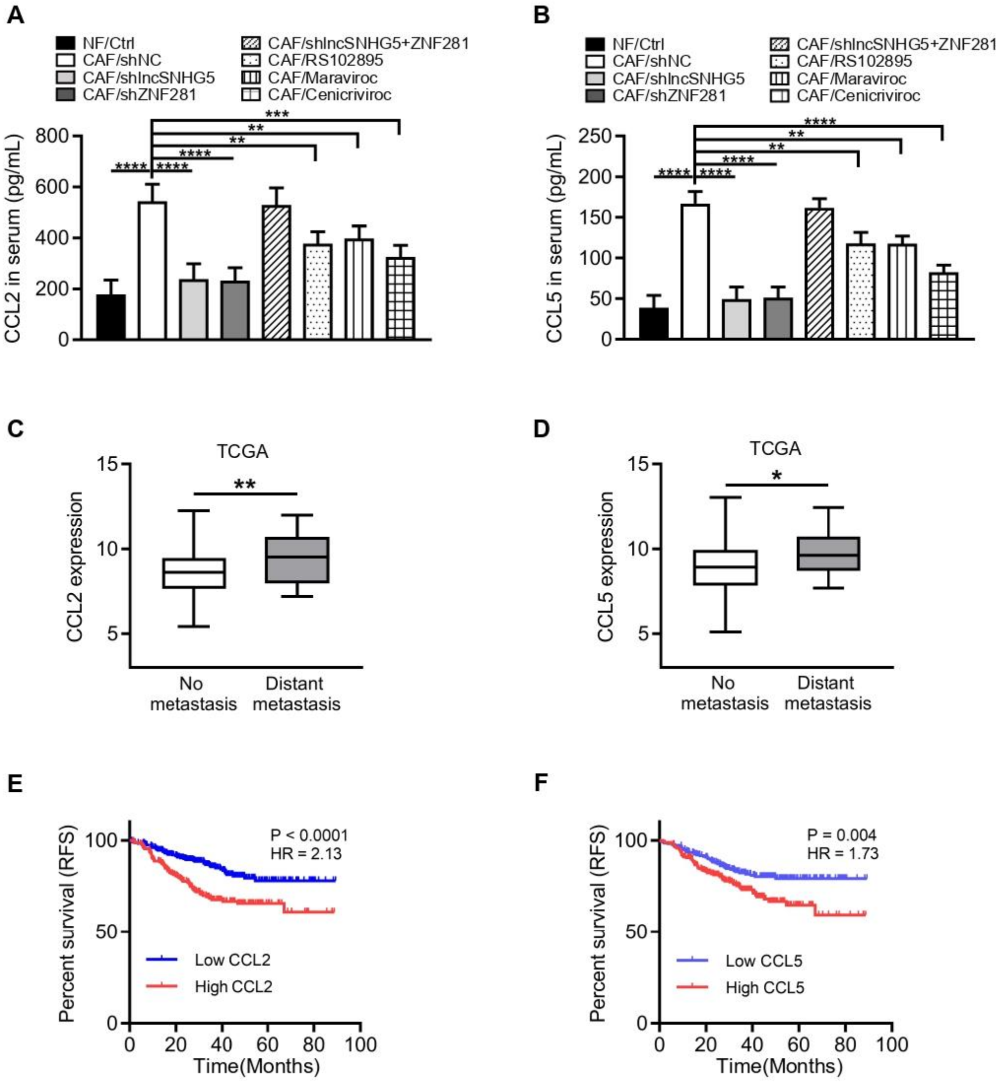

Figure S8. The lncSNHG5-ZNF281-CCL2/CCL5 signaling axis promotes BC metastasis

(A, B) Serum CCL2 and CCL5 levels in mice inoculated with NFs/Ctrl, CAFs/shNC, CAFs/shlncSNHG5, CAFs/shZNF281, CAFs/shlncSNHG5/ZNF281, CAFs/RS102895, CAFs/Maraviroc or CAFs/Cenicriviroc were analyzed by ELISA at 6 weeks after injection. (C, D) Expression levels of CCL2 (left panel) or CCL5 (right panel) in breast tumors with or without distant metastases using the TCGA dataset. (E, F) Kaplan–Meier survival analysis of relapse-free survival based on CCL2 (E) or CCL5 (F) expression using the GSE25066 dataset. Data are shown as the mean ± SD (ns: no significance, *P < 0.05, **P < 0.01, ***P < 0.001. ****P < 0.0001).

Table S1. The sequences of shRNAs

Gene names	Sequence
shNC	TTCTCCGAACGTGTCACGT
sh lncSNHG5#1	CGTTCTGAGTGTGGACGAG
sh lncSNHG5#2	GATGCAAAGATACACGAAA
shZNF281#1	GCCATGTAGTACAAGAGTAAA
shZNF281#1	GACAATGTTTAGCAATCAA
shIGF2BP2#1	AGTGAAGCTGGAAGCGCATAT
shIGF2BP2#2	TTCCCGCATCATCACTCTTAT
shCCL2#1	CCCAGTCACCTGCTGTTATAA
shCCL2#2	GCTGTGATCTTCAAGACCATT
shCCL5#1	CCACATCAAGGAGTATTTCTA
shCCL5#2	GGAGAGTCCTTGAACCTGA

Table S2

Table S2.1 Primers used for pcDNA3.1-lncSNHG5 construction

Fragment		Sequence
Full-length	FW	CTTTTACGTCGGCCTTC
	REV	TTAGTGGATTTTCCATTTAAT
1-170	FW	CTTTTACGTCGGCCTTC
	REV	GTTAAAAGTGTCAGGT
171-340	FW	AGTGAACAGCGTTCTGAGT
	REV	AACCTCGTGGCACTAGC
341-507	FW	TACTTGACTGTTGTGTGAAA
	REV	TTAGTGGATTTTCCATTTAAT

Table S2.2 Primers used for pcDNA3.1-ZNF281 construction

Fragment		Sequence
Full-length	FW	AGTACACGGGAGGCTTTTAA
	REV	TTGTGATTAACAGGACT
5'UTR	FW	AGTACACGGGAGGCTTTTAA
	REV	ACCCCGGAGGAGGCCTG
CDS	FW	ATGAAAATCGGCAGTGGGTT
	REV	TTACCTGTAACTCTGGCTGGTG
3'UTR	FW	GGTCCCAAAAGTGGCCAG
	REV	TTGTGATTAACAGGACT

Table S2.3 Primers used for pcDNA3.1-IGF2BP2 construction

Fragment		Sequence
Full-length	FW	ATGATGAACAAGCTTTACAT
	REV	TCACTTGCTGCGCTGTGAGG
RRM1/2	FW	ATGATGAACAAGCTTTACAT
	REV	TCTGGCCTGAGAAGTGCCCC
KH1/2	FW	CAGATTGATTTCCCGCTGCG
	REV	CACGGACAGTCCTGTTGAAA
KH3/4	FW	CTATCTCCACCAGCAGGGCC
	REV	CTTGCTGCGCTGTGAGGCGAC

Table S3

The sequences of primers used for qRT-PCR

Gene names	Sequence	
lncSNHG5	FW	5’-CTGAAGATGCAAAGATACACGAA-3’
	REV	5’-TTCACTGGCTACTCGTCCACA-3’
ZNF281	FW	5’-ACACGGTTTCCAATTTGTCAG-3’
	REV	5’-TAACAGATTGGCCGAAACCAC-3’
IGF2BP2	FW	5’-CAGACACAGAAACCGCCGTTG-3’
	REV	5’-TTCTCAAACTGATGCCCGCTT-3’
CCL2	FW	5’-TCAGCCAGATGCAATCAATGCC-3’
	REV	5’-GCTTCTTTGGGACACTTGCT-3’
CCL5	FW	5’-TTTCCTGTATGACTCCCGGCTGA-3’
	REV	5’-AGTTGATGTACTCCCGAACCC-3’
VEGFA	FW	5’-GAGCTTCCTACAGCACAAC-3’
	REV	5’-GATTTCTTGCGCTTTCGTT-3’
ZO-1	FW	5’-AAGGATGTTTATCGTCGCATT-3’
	REV	5’-ACAAGGTATCCACAACACGGAA-3’
Occludin	FW	5’-ATGTCATCCAGGCCTCTTGAA-3’
	REV	5’-ATACTGATCCACGTAGAGTCC-3’
β-Actin	FW	5’-TGACGTGGACATCCGCAAAG-3’
	REV	5’-CTGGAAGGTGGACAGCGAGG-3’

Table S4

Table S4.1 Primers for in vitro transcription

Gene names		Sequence
lncSNHG5 (sense)	F:	(T7)CTTTTACGTCGGCCTTCGCGAGCGTCTGGG
	R:	TTAGTGGATTTTCCATTTAATGCTCCCCAT
lncSNHG5 (antisense)	F:	TTAGTGGATTTTCCATTTAATGCTCCCCAT
	R:	(T7)CTTTTACGTCGGCCTTCGCGAGCGTCTGGG

Table S4.2 Primers for antisense oligomer affinity pull-down assays

Gene names	Sequence
lncSNHG5 sense oligo DNA	5' (biotin-) ACGTTAGACACAGCCTCCGTG
lncSNHG5 antisense oligo DNA	5' (biotin-) TGCCGAAGAGCTTCTTCTGGTT

Table S5

Table S5.1 Primers for CHIP assays

Gene names	Sequence
CCL2-1	F: GCAGAGGACTGAGACAAACAC<br>R: CTTGTTCTGCCTGAATCTCAC
CCL2-2	F: GCACAACTGAGGAATGAAGT<br>R: CTGGTTATGGCAGCTATTCTC
CCL5	F: AGGTAAAACTAAGGATGTCAGC<br>R: CTCCGGAAATTCGAGTCTCT

Table 5.2 Primers for gene-specific m6A assays

Gene names	Sequence
ZNF281	F: CTTAATCTTAAATACGCTGAGT<br>R: AAAATCATACAGCTTAAGAGA

Table S6. Correlation between the expression of lncSNHG5 and clinicopathological features in breast cancer (n=92)

Variables	Expression of lncSNHG5		Chi-square	p-value
	Low(n=46)	High(n=46)		
Age				
< 50	21	19	0.177	0.674
⩾50	25	27		
Tumor size				
⩽2 cm	20	13	2.810	0.246
2-5 cm	23	27		
> 5 cm	3	6		
Lymph node metastasis				
Yes	17	34	12.720	0.0004***
no	29	12		
Distant metastasis				
Yes	1	16	16.240	<0.0001****
no	45	30		
TNM Stage				
I/II	32	18	8.587	0.0034**
III/IV	14	28		

*p < 0.05, **p < 0.01, ***p < 0.001, ****p < 0.0001.
